# Multimodal Retinal Imaging for Systemic Microvascular Phenotyping: Chronic Kidney Disease as a Translational Reference Model

**DOI:** 10.3390/jcm15218657

**Published:** 2026-11-06

**Authors:** Qiumei Gu, Zhengju Chen, Jiacen Wan, Zhuangzhi Gao, Hongyi Qin, Ziyan He, Yalin Zheng, Fang Lu

**Affiliations:** 1 Department of Ophthalmology, West China Hospital, Sichuan University, No. 37 Guoxue Alley, Wuhou District, Chengdu 610041, China; g.qiumei@liverpool.ac.uk (Q.G.);; 2 Department of Eye and Vision Sciences, University of Liverpool, William Henry Duncan Building, 6 West Derby Street, Liverpool L7 8TX, UK; 3 Department of Radiology, Huaxi MR Research Center (HMRRC), Institute of Radiology and Medical Imaging, West China Hospital, Sichuan University, Chengdu 610041, China; chenzhengju@wchscu.cn; 4 Department of Primary Care and Mental Health, University of Liverpool, Liverpool L7 8TX, UK; 5 Institute of Life Course & Medical Sciences, University of Liverpool, Liverpool L7 8TX, UK

**Keywords:** retinal imaging, chronic kidney disease, artificial intelligence, risk enrichment, systemic microvascular phenotyping, multimodal imaging, clinical translation

## Abstract

Multimodal retinal imaging enables repeated, noninvasive assessment of vascular, neuroretinal, choroidal, perfusion, oxygenation, and flow-related phenotypes, positioning the retina as a platform for systemic microvascular phenotyping. Chronic kidney disease (CKD) provides an informative translational reference model because estimated glomerular filtration rate (eGFR) and urinary albumin-to-creatinine ratio (UACR) offer standardized clinical anchors across disease severity and progression. This critical narrative review, informed by structured searches of PubMed/MEDLINE, Embase, and Web of Science, evaluates retinal phenotypes from biological association and longitudinal prediction to incremental clinical value, external validation, and implementation. Fundus photography, ultra-widefield imaging, optical coherence tomography (OCT), OCT angiography (OCTA), and functional retinal imaging provide complementary information, but retinal signals are not organ-specific. Artificial intelligence (AI)-enabled models may support automated phenotyping and opportunistic risk enrichment, but credible translation requires appropriate comparators, external validation, calibration, subgroup assessment, decision-analytic utility, and workflow evaluation. Retinal imaging should therefore be viewed as an adjunctive platform rather than a stand-alone CKD diagnostic test, with its near-term role being to identify individuals warranting confirmatory kidney assessment when retinal images are already available and recent eGFR/UACR data are absent.

## 1. Background

Retinal imaging provides a unique opportunity to characterize microvascular and neurovascular health repeatedly and noninvasively in vivo, making the retina an increasingly attractive platform for systemic phenotyping. Chronic kidney disease (CKD) provides a particularly informative clinical model within this broader framework. In 2023, an estimated 788 million people aged 20 years or older were living with CKD, corresponding to a global age-standardized prevalence of 14.2% [1]. CKD is diagnosed and staged using estimated glomerular filtration rate (eGFR) and markers of kidney damage, particularly urinary albumin-to-creatinine ratio (UACR), interpreted according to chronicity and clinical context [2,3]. These measures are indispensable but do not directly characterize systemic microvascular structure or function; moreover, CKD spans a continuum from early kidney damage to kidney failure, with longitudinal changes measurable during disease progression and after kidney transplantation [2,3,4]. These characteristics provide standardized clinical anchors against which retinal phenotypes can be evaluated without assuming the existence of a kidney-specific retinal signature.

The retina provides high-resolution access to vascular geometry, capillary perfusion, neuroretinal and choroidal structure, and selected functional responses through complementary imaging modalities [5,6,7,8]. Importantly, however, retinal abnormalities associated with systemic disease are rarely organ-specific. Retinal phenotypes may reflect combined renal, cardiovascular, metabolic, inflammatory, ocular, treatment-related, and aging-associated influences [4,9,10,11,12]. This limited specificity is both a challenge and an opportunity: it constrains disease-specific interpretation while supporting retinal imaging as a potential platform for characterizing shared systemic microvascular injury. Cardiovascular and cardiometabolic diseases provide the strongest comparative context, whereas liver- and gut-related pathways and other neurodegenerative or immune-inflammatory conditions help define broader multi-organ influences and potential sources of heterogeneity [5,8,10,13,14].

Artificial intelligence (AI) may further extend retinal phenotyping by transforming conventional and multimodal images into automated quantitative features and risk estimates. Retinal image-based models have demonstrated the ability to detect prevalent CKD and, in selected longitudinal cohorts, estimate incident CKD risk [15,16,17,18]. In patients with diabetes, retinal models have shown potential for detecting clinically attributed diabetic kidney disease (DKD) and, in biopsy-defined cohorts, differentiating diabetic nephropathy (DN) from non-diabetic kidney disease (NDKD) [19]. Nevertheless, discrimination alone does not establish clinical usefulness. Translation requires external validation, calibration, fairness assessment, clinically interpretable outputs, decision-curve analysis, and prospective workflow evaluation [20,21,22,23,24,25,26]. Given the shared nature of retinal microvascular signals, the most credible near-term role of these approaches is systemic risk enrichment and decision support rather than stand-alone organ-specific diagnosis.

Previous work has established the broader concept of retinal imaging and oculomics as windows into systemic health [8,27], summarized retinal manifestations of kidney disease [6,7,28], examined shared small-vessel abnormalities across the brain, retina, and kidney [29], and reviewed AI-enabled retinal oculomics [30,31]. Taken together, these contributions leave a complementary translational question insufficiently addressed: how multimodal retinal phenotypes progress from biological association and longitudinal prediction to incremental clinical value, external validation, and implementation, while explicitly accounting for the limited organ specificity of shared retinal signals.

Accordingly, this critical narrative review uses CKD as a clinically anchored reference model to evaluate the translational value of multimodal retinal imaging. We focus on five questions: the biological rationale for shared retinal and renal microvascular abnormalities; the retinal imaging phenotypes associated with CKD; the strength of longitudinal and incremental predictive evidence; the contribution of AI-enabled risk models; and the evidence required before clinical implementation. Diabetes, hypertension, and cardiovascular disease are considered primarily to distinguish kidney-associated findings from shared cardiometabolic microvascular burden. The central question is whether retinal information improves a defined clinical decision beyond available clinical and laboratory assessment.

### Literature Search and Evidence Synthesis

This critical narrative review was informed by structured searches of PubMed/MEDLINE, Embase (Ovid), and Web of Science Core Collection from database inception through 27 July 2026, supplemented by targeted searches and reference-list screening updated through 31 July 2026. Evidence was prioritized according to direct relevance, methodological informativeness, longitudinal or external validation, and translational utility. This review was not designed as a systematic review or meta-analysis. Full search strategies, record counts, evidence-selection criteria, and the evidence-selection framework are provided in Supporting Information S1. Narrative-review transparency was informed by the Scale for the Assessment of Narrative Review Articles (SANRA) framework [32].

## 2. Biological Basis: CKD as a Translational Reference Model for Shared Systemic Microvascular Injury

**Figure 1:**
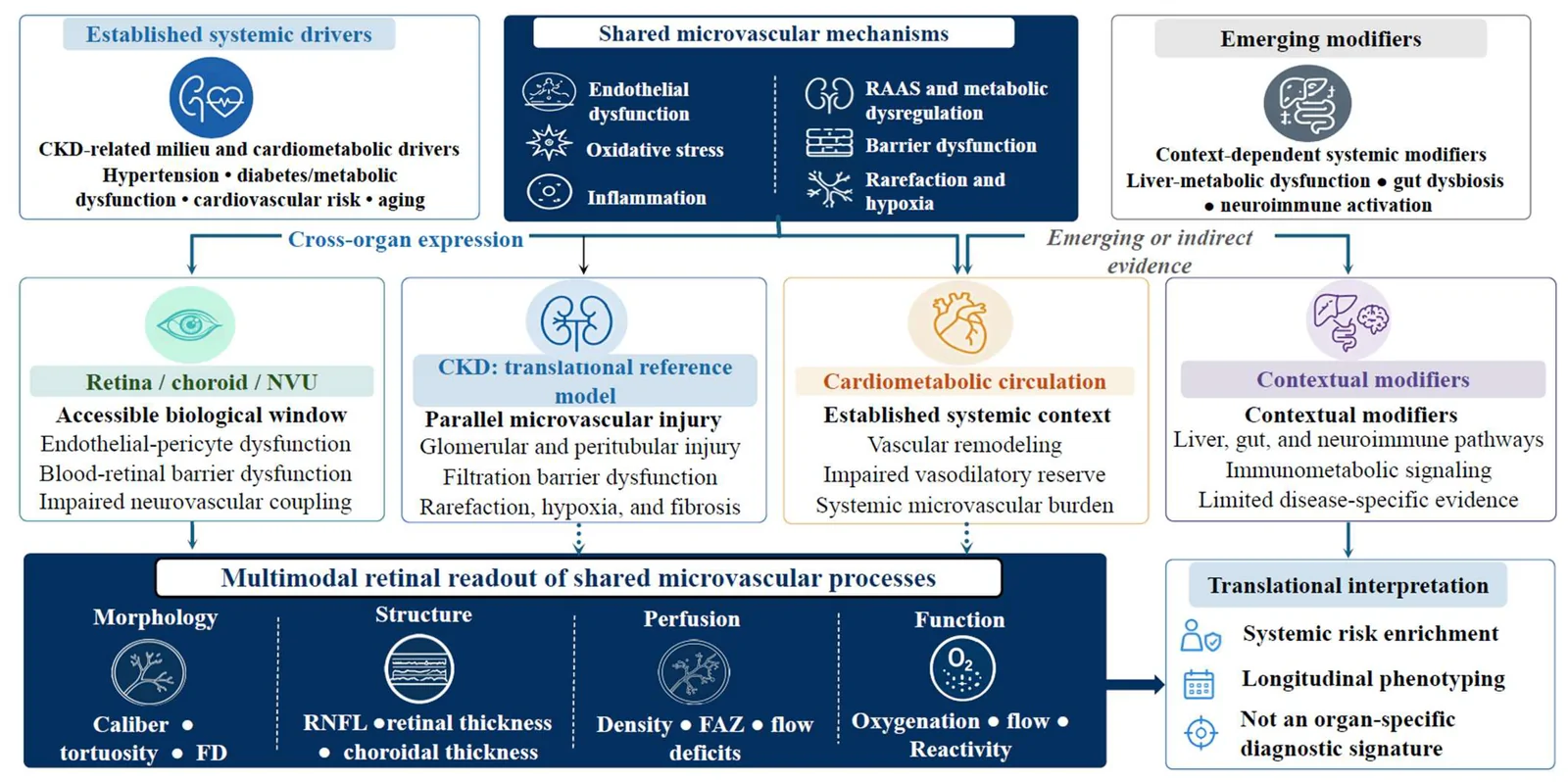
**Mechanistic framework for shared systemic microvascular influences on retinal phenotypes, with CKD as a translational reference model.** Established systemic drivers and emerging contextual modifiers converge on shared microvascular mechanisms that may produce parallel but non-equivalent patterns of retinal, renal, and cardiometabolic injury. Solid pathways indicate comparatively established biological or clinical relationships, whereas dashed connections indicate contextual, emerging, or indirect relationships. Retinal phenotypes should be interpreted as adjunctive markers for systemic risk enrichment and longitudinal phenotyping, not as organ-specific diagnostic signatures or evidence of causal kidney–eye pathways. CKD, chronic kidney disease; FAZ, foveal avascular zone; FD, fractal dimension; NVU, neurovascular unit; RAAS, renin–angiotensin–aldosterone system; RNFL, retinal nerve fiber layer.

Systemic diseases may influence the retina and kidney through convergent hemodynamic, metabolic, endothelial, and immune pathways. This review therefore uses CKD as a translational reference model for shared microvascular and neurovascular injury. Cardiometabolic disease provides the best-established comparative context, while liver-, gut-, neurodegenerative-, and immune-inflammatory factors are treated as exploratory contextual modifiers that may explain heterogeneity rather than as parallel diagnostic targets (Figure 1).

### 2.1. Shared Endothelial and Microvascular Injury Pathways

Endothelial dysfunction links retinal abnormalities to CKD and cardiometabolic disease. Reduced nitric oxide bioavailability, oxidative stress, inflammation, renin–angiotensin–aldosterone system (RAAS) activation, and metabolic dysregulation disrupt vascular tone, barrier integrity, and perfusion, promoting capillary rarefaction and hypoxia [6,10,33,34]. These processes contribute to renal microvascular injury and may manifest in the retina as alterations in vascular geometry, capillary perfusion, oxygenation, and neural or choroidal structure [4,9,11,12].

### 2.2. Structural and Functional Parallels Between Retinal and Renal Microcirculations

The retinal and renal microcirculations are comparable but not anatomically equivalent. Both are metabolically active, autoregulated vascular beds with specialized barriers, but their barrier architecture and flow regulation differ substantially [6,7,34]. Both remain vulnerable to injury from diabetes, hypertension, inflammation, and oxidative stress and may consequently undergo capillary rarefaction. Associations of retinal geometry, optical coherence tomography angiography (OCTA) perfusion, and retinal or choroidal structure with albuminuria, eGFR, incident CKD, and CKD progression support biomarker evaluation, but heterogeneity in study populations, CKD etiologies, ocular conditions, and imaging devices precludes their use as surrogates for renal pathology [4,9,12,35].

### 2.3. Neurovascular Unit Dysfunction and Systemic Microvascular Disease

The retinal neurovascular unit (NVU) comprises vascular, neural, glial, immune, and extracellular matrix components that coordinate barrier integrity, metabolic exchange, and activity-dependent blood flow [6,36,37]. Systemic metabolic, hemodynamic, and inflammatory stress can disrupt this crosstalk, causing leakage, impaired reactivity, capillary nonperfusion, gliosis, and neuronal injury. Concurrent structural and perfusion abnormalities reported in CKD may therefore represent composite vascular–neural–glial responses rather than isolated capillary injury [4,12,37,38].

The kidney does not contain a retinal-type NVU. Its microvascular integrity nevertheless depends on coordinated interactions among glomerular endothelial cells, podocytes, mesangial cells, tubular epithelial cells, pericytes, immune cells, and the extracellular matrix [6,34]. This represents a functional analogy rather than anatomical homology. In both organs, disruption of multicellular microvascular interactions may contribute to barrier failure, impaired perfusion, hypoxia, inflammation, and remodeling.

Multimodal retinal imaging can interrogate complementary aspects of this response, including vascular and structural changes, capillary perfusion, oxygenation, blood-flow dynamics, and vascular reactivity. Concordant abnormalities may provide a more coherent phenotype than any single parameter, but direct evidence that CKD causes retinal NVU dysfunction remains limited; these findings should not be interpreted as proof of a causal kidney–eye pathway.

### 2.4. Cardiometabolic–Eye Axis and Contextual Modifiers

The cardiometabolic–eye axis is the best-established extension of this framework. Hypertension, diabetes, obesity, dyslipidemia, and cardiovascular disease share endothelial dysfunction, oxidative stress, inflammation, vascular stiffness, and impaired autoregulation [5,10,34,39]. Hypertensive and glycemic exposures both contribute to CKD and independently alter retinal geometry, perfusion, and neural or choroidal structure; retinal findings should therefore be interpreted in the context of systemic risk factors rather than attributed to a single organ.

Metabolic dysfunction-associated steatotic liver disease (MASLD) and gut-related inflammation may further modify this cardiometabolic milieu through insulin resistance, oxidative stress, endothelial dysfunction, microbial metabolites, and immune signaling [10,14,40,41]. Current evidence supports treating these factors as contextual covariates or effect modifiers rather than as independent retinal screening targets (Table 1).

## 3. Multimodal Ophthalmic Imaging Biomarkers

**Figure 2:**
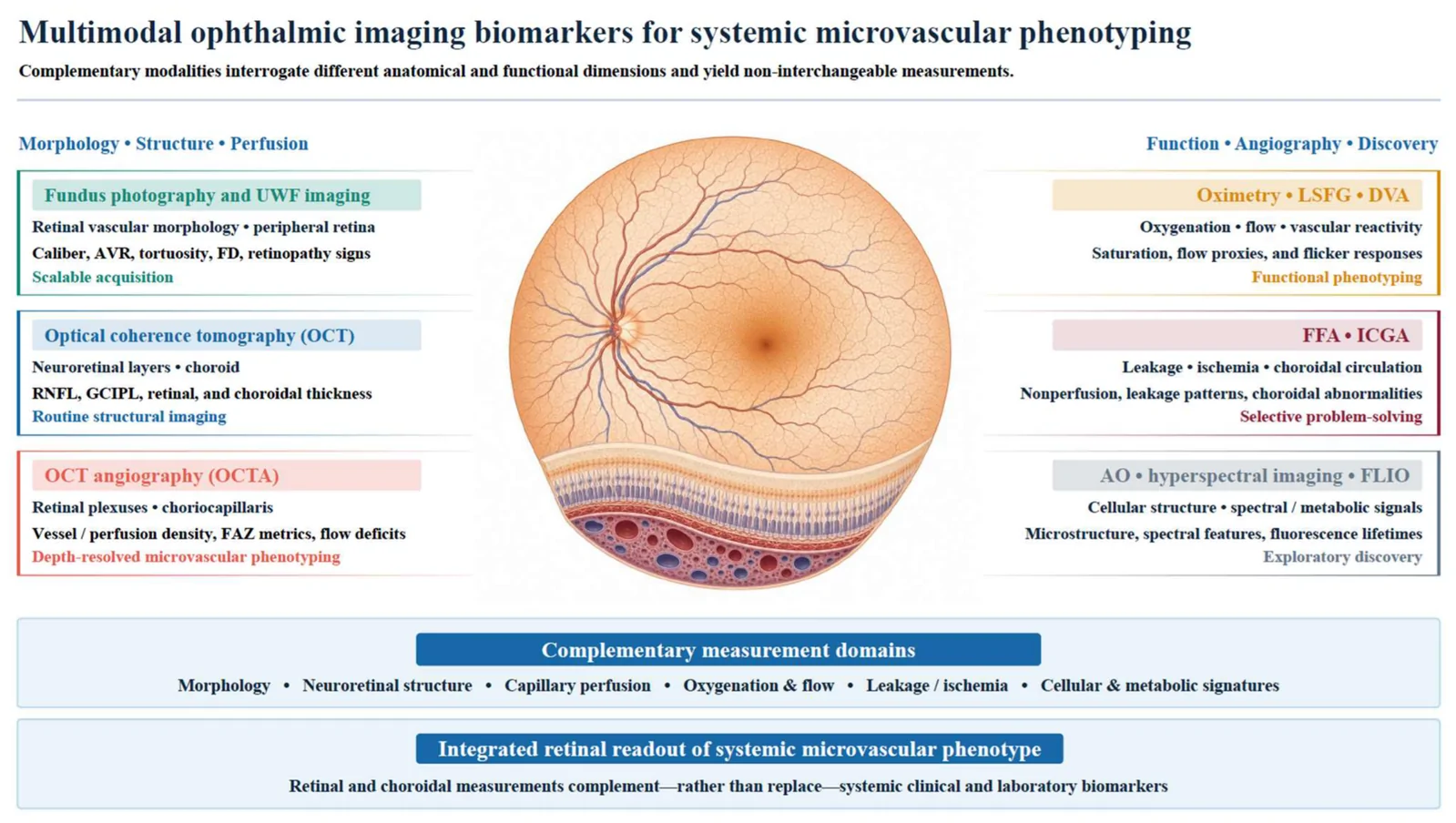
**Multimodal ophthalmic imaging biomarkers for systemic microvascular phenotyping.** Complementary ophthalmic imaging modalities characterize distinct dimensions of retinal and choroidal morphology, neuroretinal structure, capillary perfusion, oxygenation, blood flow dynamics, vascular reactivity, leakage, ischemia, and cellular or metabolic signatures. FP and UWF imaging support scalable assessment of retinal vascular morphology and peripheral retinal features; OCT and OCTA provide structural and depth-resolved microvascular phenotyping; retinal oximetry, LSFG, and DVA assess functional alterations; FFA and ICGA enable targeted evaluation of leakage, nonperfusion, and choroidal circulation; and AO, hyperspectral imaging, and FLIO remain primarily exploratory. Together, these complementary measurements provide an integrated retinal readout of systemic microvascular phenotypes, but the translational positions indicated in the figure are contextual rather than formal evidence grades. Retinal measurements should therefore be interpreted alongside systemic clinical and laboratory biomarkers. AO, adaptive optics; AVR, arteriolar-to-venular ratio; DVA, dynamic vessel analysis; FAZ, foveal avascular zone; FD, fractal dimension; FFA, fundus fluorescein angiography; FLIO, fluorescence lifetime imaging ophthalmoscopy; FP, fundus photography; GCIPL, ganglion cell–inner plexiform layer; ICGA, indocyanine green angiography; LSFG, laser speckle flowgraphy; OCT, optical coherence tomography; OCTA, optical coherence tomography angiography; RNFL, retinal nerve fiber layer; UWF, ultra-widefield imaging.

Each imaging modality captures a different component of the systemic microvascular phenotype; no single measure is interchangeable with another, nor is any measure specific to CKD (Figure 2). Fundus photography characterizes vascular morphology and retinopathy signs; optical coherence tomography (OCT) assesses retinal and choroidal structure; OCTA provides depth-resolved microvascular flow signals; and functional techniques add information on oxygenation, blood-flow dynamics, and vascular reactivity.

### 3.1. Fundus Photography and Ultra-Widefield Imaging

Color fundus photography (FP) is the most scalable modality for automated assessment of vessel caliber, arteriolar-to-venular ratio (AVR), tortuosity, fractal dimension (FD), branching geometry, and retinopathy [8,9,45,46]. These features have been associated with CKD and cardiometabolic outcomes but remain sensitive to demographic, vascular, ocular, image-quality, and analytic factors; they indicate systemic microvascular burden rather than kidney-specific injury [9,35,39,42,47].

Ultra-widefield (UWF) imaging expands retinal coverage and can reveal peripheral vascular or retinopathy lesions that may be missed by conventional central-field photography. This wider field of view is clinically valuable in retinal diseases with diagnostically or prognostically relevant peripheral involvement; for example, predominantly peripheral diabetic retinopathy lesions have been associated with subsequent disease progression [46]. In CKD, a multicenter study reported modestly higher discrimination for a UWF–based model than for a central-region model, both internally and in multicenter testing, suggesting that peripheral retinal information may improve discrimination compared with central-field images [18]. However, peripheral vessel annotation and segmentation are technically more difficult, and current CKD evidence remains largely cross-sectional, device-specific, and geographically limited. UWF imaging should therefore be regarded as a potentially informative extension of FP rather than an established requirement for CKD screening.

### 3.2. OCT and OCTA

OCT provides high-resolution structural assessment of the neuroretina and choroid, including thickness measurements of the retinal nerve fiber layer (RNFL), ganglion cell–inner plexiform layer (GCIPL), macula, and choroid. Reduced inner retinal thickness and choroidal alterations have been reported in CKD and may reflect neurovascular vulnerability, chronic hypoxia, fluid-related changes, or systemic vascular remodeling [4,38,48,49]. In a cross-cohort analysis, lower baseline eGFR was associated with thinner GCIPL in 23,014 UK Biobank participants and with faster GCIPL loss over approximately three years in 2197 Chinese participants; relative to eGFR ≥ 90 mL/min/1.73 m^2^, the fully adjusted annual excess thinning was 0.30 μm for eGFR 60–89 and 0.52 μm for eGFR < 60 [50]. In 1408 ocular treatment-naive adults with type 2 diabetes, lower eGFR was also associated with lower peripapillary choroidal thickness and selected peripapillary retinal nerve fiber layer (pRNFL) thickness measures, while microalbuminuria was inversely associated with average peripapillary choroidal thickness [51]. A separate 2-year cohort found faster pRNFL loss in 152 CKD participants than in 40 controls (−0.87 versus −0.26 μm/year), with progressively greater loss from G3a to kidney failure [52]; the single-center design and 22% attrition limit generalizability. Together, these observational studies broaden retinal biomarker research beyond vascular morphology alone without proving kidney-specific neurodegeneration.

OCTA provides dye-free, depth-resolved flow-related measures such as vessel or perfusion density, foveal avascular zone (FAZ) morphology, and choriocapillaris flow deficits [4,12,53,54,55,56,57], but it neither quantifies absolute blood flow nor demonstrates leakage. Measurements are influenced by signal strength, scan size, segmentation boundaries, motion and projection artifacts, axial length, diurnal variation, test–retest variability, and proprietary device algorithms; deep-capillary-plexus metrics are particularly vulnerable to segmentation and projection errors [54,56,58,59]. Small between-group differences should therefore be interpreted alongside device- and protocol-specific repeatability and potential systematic differences in image quality or segmentation [58,59]. Statistical significance at the group level does not establish reliable individual-level discrimination. Similarly, small within-person changes require comparison with measurement variability before being interpreted as biological progression or treatment response.

### 3.3. Retinal Oximetry, LSFG, and Dynamic Vessel Analysis

Retinal oximetry, laser speckle flowgraphy (LSFG), and dynamic vessel analysis (DVA) add functional information on oxygenation, relative blood-flow signals, and vascular reactivity [11,60,61]. In an age-matched analysis, hemodialysis patients had attenuated flicker-induced arteriolar and venular dilation compared with healthy controls [62], although substantial between-group differences in diabetes, hypertension, inflammatory status, and vascular disease burden preclude kidney-specific attribution. Associations between retinal vascular responses and mortality in dialysis populations further support biological relevance [63], but sensitivity to measurement conditions, limited reference ranges, and uncertain incremental value currently restrict these methods to complementary research applications.

### 3.4. FFA and ICGA

Fundus fluorescein angiography (FFA) demonstrates leakage, nonperfusion, ischemia, and neovascularization, while indocyanine green angiography (ICGA) visualizes the choroidal circulation [46]. Intravenous dye and limited CKD-specific evidence make both selective ocular problem-solving tools rather than platforms for population risk enrichment.

### 3.5. Emerging Technologies: Adaptive Optics, Hyperspectral Imaging, and FLIO

Adaptive optics offers near-cellular visualization, hyperspectral imaging captures wavelength-resolved tissue signatures, and fluorescence lifetime imaging ophthalmoscopy (FLIO) measures endogenous fluorescence lifetimes [64,65,66]. Evidence for their systemic and CKD applications remains preliminary; translation requires standardized protocols, normative data, repeatability, external validation, and added value beyond established imaging and clinical biomarkers.

Across modalities, three practical translational positions can be distinguished. Conventional fundus photography is the most scalable platform for prospective evaluation of opportunistic CKD risk enrichment, with UWF imaging offering an extension where equipment is available [16,17,18,19]. Quantitative OCT/OCTA measures and functional assessments remain research applications for CKD: selected OCT/OCTA measures have emerging longitudinal evidence, DVA has prognostic evidence in dialysis populations, and retinal oximetry and LSFG provide complementary physiological information [4,12,50,52,60,61,62,63]. Adaptive optics, hyperspectral imaging, and FLIO remain exploratory for CKD-related applications [64,65,66]. These positions reflect practical availability, standardization, reproducibility, resource requirements, and CKD-specific evidence rather than formal evidence grades. Established ophthalmic use does not itself establish readiness for CKD risk assessment; FFA and ICGA retain selective ocular indications rather than a role in routine opportunistic kidney-risk assessment.

## 4. Clinical Evidence: CKD as a Translational Reference Model

**Figure 3:**
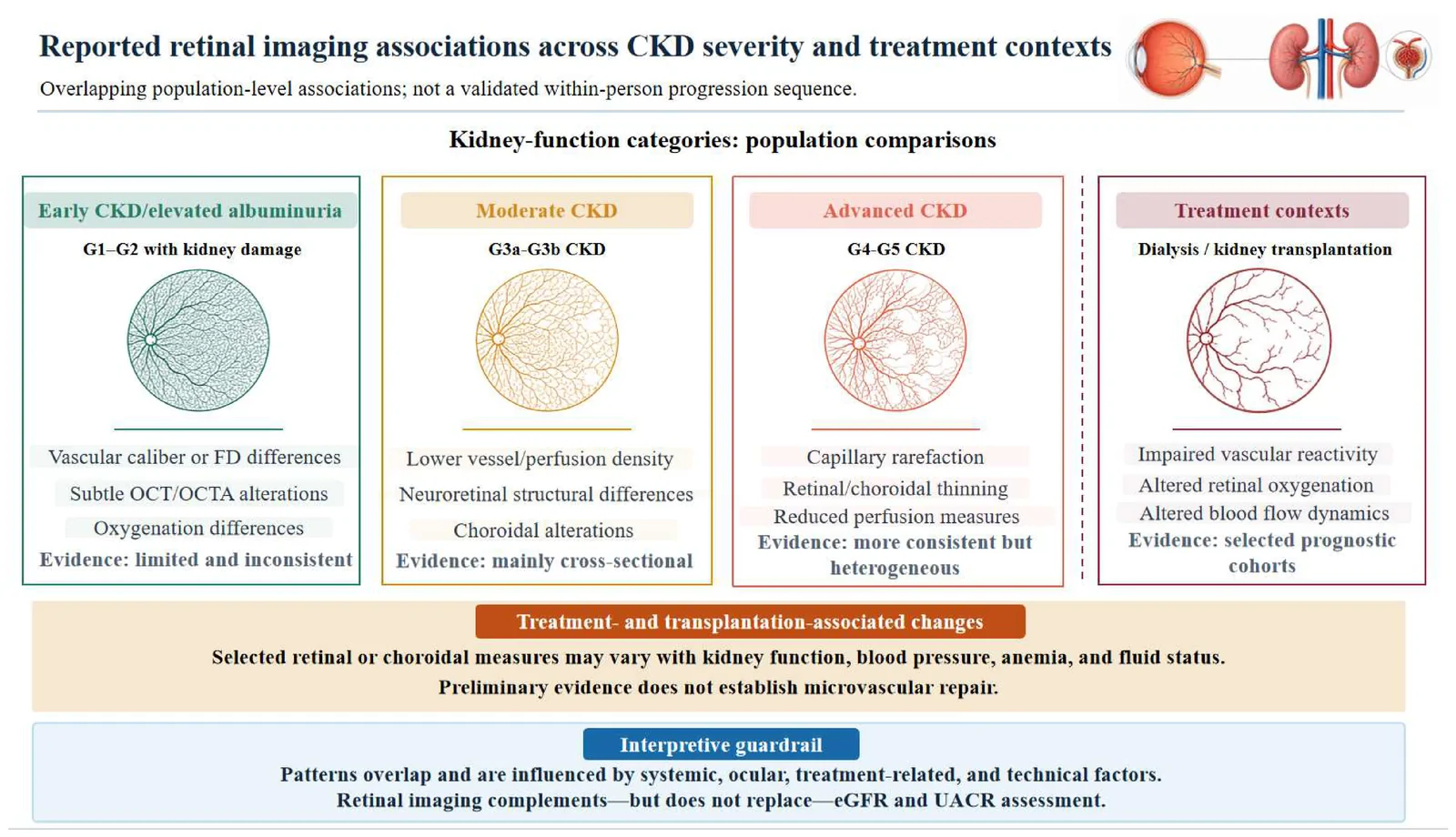
**Reported retinal imaging associations across CKD severity and treatment contexts.** This schematic summarizes overlapping population-level findings from heterogeneous studies, predominantly cross-sectional. Features are not exclusive to individual kidney-function categories, and the panel order does not represent a validated retinal staging system, within-person progression sequence, or causal kidney-to-retina pathway. Dialysis and transplantation are shown separately as treatment contexts rather than successive stages on an eGFR continuum. Treatment-associated changes remain preliminary and should not be interpreted as established microvascular repair. CKD, chronic kidney disease; eGFR, estimated glomerular filtration rate; FD, fractal dimension; OCT, optical coherence tomography; OCTA, optical coherence tomography angiography; UACR, urinary albumin-to-creatinine ratio.

CKD permits evaluation of retinal phenotypes in relation to kidney function, albuminuria, disease severity, prognosis, and treatment-related changes [3,4,9]. Table 2 summarizes representative studies. Figure 3 summarizes overlapping population-level retinal associations reported across kidney-function categories and treatment contexts. Features shown in different groups are not exclusive to those groups, and their arrangement does not imply a validated within-person progression sequence.

### 4.1. Cross-Sectional Evidence

Cross-sectional studies constitute the largest body of evidence linking retinal abnormalities with CKD. A systematic review of 42 studies identified associations between retinopathy, retinal vascular caliber, and several kidney outcomes, including prevalent CKD, albuminuria, kidney failure, and eGFR decline [9]. Population cohorts have linked venular tortuosity and reduced vascular network complexity with impaired kidney function [67,68], but in the Singapore Malay Eye Study (SiMES), retinal geometry and qualitative signs were associated with continuous eGFR or albuminuria, whereas none independently identified prevalent CKD as a binary outcome; adding retinal markers improved the area under the receiver operating characteristic curve (AUC) for microalbuminuria from 0.77 to 0.80 but not for CKD [69]. Scanning laser Doppler flowmetry has also identified arteriolar remodeling and capillary rarefaction in moderately severe CKD [70]. In diabetes, albuminuria was associated with diabetic retinopathy, whereas isolated eGFR < 60 mL/min/1.73 m^2^ without albuminuria was not associated with diabetic retinopathy; the combined low-eGFR/albuminuric phenotype had the strongest association [71]. This heterogeneity cautions against treating all reduced-eGFR phenotypes as equivalent manifestations of diabetic microangiopathy.

OCTA studies, including hypertensive and mixed CKD cohorts, have reported lower retinal vessel density and larger flow-void areas [12,72,73,74]. OCT studies provide complementary evidence of inner retinal and choroidal thinning, although some abnormalities are more apparent in advanced disease than in early CKD [4,38]. Not all cohorts have demonstrated consistent associations, highlighting the effects of diabetes, hypertension, age, ocular disease, imaging protocol, and statistical adjustment [75].

### 4.2. Longitudinal and Prognostic Evidence

Longitudinal evidence is less extensive but more clinically informative. In SiMES, smaller baseline retinal arterioles, larger venules, and retinopathy were associated with incident CKD over a median follow-up of six years [35]. In two additional Asian cohorts, retinopathy predicted incident kidney failure (reported as end-stage renal disease [ESRD]; adjusted hazard ratio (HR) 2.51), whereas caliber and FD did not; only 33 events occurred and most were in participants with diabetes [76]. The Multi-Ethnic Study of Atherosclerosis found no overall association between retinal caliber and incident G3 CKD, with an association for arteriolar narrowing confined to White participants [77]. Over 15 years in Beaver Dam, baseline caliber did not predict incident low eGFR, and low eGFR did not predict subsequent caliber change [78]. In the Cardiovascular Health Study, retinopathy was associated with creatinine and eGFR deterioration [79], but retinal photography overlapped the kidney-function observation window. In multiethnic diabetes populations, retinal microvascular signs have been linked to diabetic kidney disease, but the clinical contribution beyond conventional risk factors requires evaluation against prespecified comparators [42]. These findings support risk enrichment while showing that an association does not necessarily translate into clinically meaningful incremental predictive value.

Prospective evidence spans vascular morphology, vascular reactivity, OCTA perfusion, and OCT-derived structure. Among 906 people with type 2 diabetes but no diabetic retinopathy, lower baseline superficial and radial peripapillary capillary perfusion densities at the optic nerve head predicted faster eGFR decline over three years; adding OCTA increased AUC from 0.696 to 0.725, with directionally consistent findings in a second 400-person cohort from the same project rather than in an independent external validation cohort [80]. In a cardiovascular-risk cohort, attenuated flicker-induced arteriolar dilation was associated with faster long-term eGFR decline among participants with initially preserved kidney function [81]. In a non-diabetic population, wider retinal arteriolar and venular calibers were associated with steeper decline in measured glomerular filtration rate (GFR) over a median 11-year follow-up [82]. Lower macular volume and choroidal thickness have also been associated with subsequent eGFR decline [4]. In kidney failure, impaired flicker-induced retinal venular dilation independently predicted all-cause mortality in a multicenter dialysis cohort [63]; this finding reflects systemic vascular prognosis rather than prediction of CKD onset or stage.

**Table 2:** **Representative clinical evidence linking retinal imaging phenotypes with CKD.**

Study	Design/ Population	Imaging Modality and Retinal/Choroidal Phenotype	Kidney or Clinical Outcome	Main Finding	Validation and Incremental Value	Clinical Utility and Key Limitations
Aronov et al. 2021 [9]	Systematic review/meta-analysis; 42 studies: 9 prospective, 32 cross-sectional, 1 retrospective	Mainly FP; retinopathy, vessel caliber, vascular signs	CKD, albuminuria, kidney failure, eGFR decline	Retinal vascular signs were associated with several kidney outcomes	Association/prognostic synthesis; not prediction-model validation.	Heterogeneous populations, definitions, and methods
Yip et al. 2017 [35]	Prospective SiMES cohort; 1256 adults without baseline CKD; median follow-up 6 years	FP/SIVA; caliber, tortuosity, FD, branching angle, retinopathy	Incident eGFR < 60 mL/min/1.73 m^2^ with ≥25% reduction	HRs: 1.34 per SD decrease in CRAE; 2.35 for highest versus lowest CRVE tertile; 2.54 for retinopathy.	Single-cohort longitudinal associations; incremental prediction not established.	Incident cases predominantly had diabetes (70.5%) or hypertension (94.9%).
Peng et al. 2020 [73]	Case–control; 256 CKD G3–G5 patients and 70 age-matched controls	OCTA; SVP/DVP density, SVP flow-void area	CKD status; preceding-year blood-pressure exposure	CKD was associated with lower density and larger SVP flow-void area; systolic blood-pressure indices correlated with SVP injury.	Cross-sectional association; not longitudinal prediction.	Shared vascular exposures; temporal relationships and treatment response unresolved.
Paterson et al. 2020 [38]	Cross-sectional cohort; 241 consented to OCT; 223 had measurable images	SD-OCT/FP; retinal layers, microvascular and choroidal measures	eGFR; source-defined CKD categories	Inner retinal thinning and microvascular variation were associated with advanced CKD, without support for early-stage detection.	Cross-sectional association; not longitudinal prediction or model validation.	Substantial comorbidity; reference-category eGFR did not establish chronicity.
Yip et al. 2015 [76]	Pooled cohorts; *n* = 5763; 21 prevalent kidney-failure cases excluded from longitudinal analysis; median follow-up 4.3 years	FP; retinopathy, CRAE/CRVE, FD	Registry-confirmed incident kidney failure, reported as ESRD	33 events; adjusted HR for retinopathy 2.51 (95% CI 1.14–5.54); caliber and FD were not associated.	Longitudinal association; incremental discrimination and external model validation not established.	Few events, mainly in diabetes; albuminuria unavailable.
Günthner et al. 2019 [63]	Multicenter prospective ISAR cohort; 214 hemodialysis patients; mean mortality follow-up 44 months	DVA; maximum flicker-induced arteriolar/venular dilation	All-cause and cardiovascular mortality; combined cardiovascular events	Greater vMax was associated with lower all-cause mortality: adjusted HR per SD 0.65 (95% CI 0.47–0.91). Cardiovascular-event associations did not persist after adjustment.	Adding vMax to age, vascular comorbidities, catheter use, diabetes, and creatinine yielded 3-year mortality NRI of 0.37 (categorical) and 0.27 (continuous). Within-cohort analysis; external validation not reported in the reviewed main text.	Imaging-guided benefit not evaluated; few events; threshold-sensitive NRI; 25.4% excluded for inadequate data quality.
Farrah et al. 2023 [4]	Cross-sectional/longitudinal studies; prognostic CKD cohort *n* = 262; transplant cohort *n* = 25	SD-OCT; macular volume, retinal/choroidal thickness	Kidney scarring, eGFR decline, transplantation/donation-related changes	Lower macular volume and temporal choroidal thickness were associated with ≥10% eGFR decline at 1 year; corresponding fully adjusted 2-year associations were nonsignificant. Choroidal thickness increased after transplantation.	Longitudinal association/treatment-context responsiveness; incremental prediction not established.	Single center; diabetes excluded; small transplant cohort; imaging-guided benefit unestablished.
Liu et al. 2025 [53]	Cross-sectional; 1050 participants with type 2 diabetes	SS-OCT/SS-OCTA; SCP/DCP density, macular choroidal thickness	Creatinine–cystatin C-based eGFR	Lower eGFR was associated with lower SCP density/choroidal thickness, but higher DCP density; patterns differed by DR status.	Cross-sectional association; sensitivity/stratified analyses, not independent model validation.	Associations vary by retinal layer and DR status; these findings do not establish clinical utility.
Huang et al. 2023 [80]	Three-year prospective GDES cohort; 906 adults with type 2 diabetes without DR; second subcohort *n* = 400	ONH OCTA; SCP/RPC perfusion; 3 × 3-mm and 6 × 6-mm scans, respectively	Annual eGFR slope; lowest versus highest tertiles	Lower baseline perfusion was associated with faster decline; adding ONH perfusion increased AUC from 0.696 to 0.725 (*p* = 0.031).	Beyond eGFR, HbA1c, and diabetes duration: NRI 0.464; IDI 0.0321 (both *p* < 0.001). Second subcohort replicated associations, not independent external model validation.	Extreme-tertile outcome; restricted population/device setting; imaging-guided benefit not evaluated.
Guo et al. 2024 [50]	Cross-cohort; UKB: *n* = 23,014, cross–sectional; COIP: *n* = 2197, longitudinal	OCT; GCIPL thickness and annual thinning	Kidney-function categories; subsequent retinal change	GCIPL thinning was faster by 0.30/0.52 μm/year in participants with eGFR 60–89/<60 mL/min/1.73 m^2^, respectively, versus ≥90.	Cross-cohort associations and longitudinal retinal change; not incident-CKD prediction or external model validation.	CKD chronicity requires qualification; causality unestablished.
Edwards et al. 2005 [79]	CHS cohort; 1394 older adults with retinal/creatinine data	FP; retinopathy, arteriolar abnormalities	Four-year creatinine/eGFR change	Retinopathy was associated with creatinine increase ≥0.3 mg/dL (OR 3.20, 95% CI 1.58–6.50) and eGFR decline ≥20% (OR 2.84, 95% CI 1.56–5.16); arteriolar abnormalities were not.	Association with kidney-function deterioration; incremental prediction not established.	Photography overlapped the kidney observation window, limiting baseline prognostic interpretation.
Liu et al. 2020 [51]	Cross-sectional study; 1408 ocular treatment-naïve adults with type 2 diabetes	SS-OCT; peripapillary choroidal/RNFL thickness	Xiangya equation-derived eGFR	Average pCT and nasal pRNFL thickness were positively associated with eGFR after adjustment (β = 0.3/0.1, respectively).	Cross-sectional structural association; not longitudinal prediction.	Single-time-point assessment; eGFR categories alone do not establish chronicity.
Sabanayagam et al. 2014 [71]	Cross-sectional SP2 *n* = 301; independent SiMES replication *n* = 265	FP; any/moderate DR	DR by eGFR/albuminuria category	Any-DR ORs: 2.33 for albuminuria with normal eGFR; 1.38 for isolated low eGFR; 2.64 for low eGFR with albuminuria. Isolated low eGFR was not significantly associated with DR.	Independent association replication; not external prediction-model validation.	DR was the dependent outcome; not evidence of retinal prediction of CKD.
Betzler et al. 2022 [83]	Prospective multiethnic SEED cohort; incident-DKD analysis *n* = 1199; approximately 6-year follow-up	FP; DR and retinal vascular profiling	Incident DKD; 175 events	DR was associated with incident DKD (RR 1.44, 95% CI 1.06–1.96); vascular profiling added limited predictive information.	AUC/NRI assessed beyond traditional risk factors; largest gains concerned hypertension, not DKD. Independent model validation could not be confirmed from accessible material.	Outcome-dependent gains; retinal-guided clinical benefit unestablished.

**Note:** Studies illustrate evidence across imaging modalities, kidney phenotypes, and study designs. Selection was representative rather than exhaustive; see Supporting Information S1, Section 3 and Section 4. Associations or improvements in discrimination or reclassification alone do not establish clinical utility. **Abbreviations:** AUC, area under the receiver operating characteristic curve; CHS, Cardiovascular Health Study; COIP, Chinese Ocular Imaging Project; CRAE, central retinal arteriolar equivalent; CRVE, central retinal venular equivalent; DCP, deep capillary plexus; DKD, diabetic kidney disease; DR, diabetic retinopathy; DVP, deep vascular plexus; eGFR, estimated glomerular filtration rate; ESRD, end-stage renal disease (retained only when reporting terminology used in the source studies); FD, fractal dimension; FP, fundus photography; GCIPL, ganglion cell–inner plexiform layer; HR, hazard ratio; OCT, optical coherence tomography; OCTA, optical coherence tomography angiography; OR, odds ratio; pCT, peripapillary choroidal thickness; pRNFL, peripapillary retinal nerve fiber layer; RR, risk ratio; RVP, retinal vascular profile; SCP, superficial capillary plexus; SD, standard deviation; SD-OCT, spectral-domain optical coherence tomography; SEED, Singapore Epidemiology of Eye Diseases; SiMES, Singapore Malay Eye Study; SIVA, Singapore I Vessel Assessment; SP2, Singapore Prospective Study Program; SS-OCT, swept-source optical coherence tomography; SS-OCTA, swept-source optical coherence tomography angiography; SVP, superficial vascular plexus; UACR, urinary albumin-to-creatinine ratio; UKB, UK Biobank; DVA, dynamic vessel analysis; GDES, Guangzhou Diabetic Eye Study; IDI, integrated discrimination improvement; ISAR, Risk Stratification in End-Stage Renal Disease; NRI, net reclassification improvement; ONH, optic nerve head; RPC, radial peripapillary capillary; vMax, maximum flicker-induced venular dilation; CI, confidence interval; HbA1c, glycated hemoglobin.

### 4.3. Differences Across CKD Stages

Current studies suggest an overall severity–related gradient, but no reproducible stage-specific retinal signatures. Findings among participants with eGFR 60–89 mL/min/1.73 m^2^ or other early-risk features are subtle and inconsistent and may include differences in vascular caliber, OCT/OCTA measures, FD, or oxygenation. An eGFR of 60–89 mL/min/1.73 m^2^ alone does not establish CKD; evidence of kidney damage persisting for at least 3 months is required. Studies without this documentation describe a G2-range kidney-function phenotype rather than confirmed G2 CKD. Their ability to distinguish early CKD from cardiometabolic risk without kidney disease remains uncertain. In G3 CKD, reductions in OCTA-derived vessel or perfusion density and early retinal or choroidal alterations are reported more consistently [73,84]. Advanced G4–G5 CKD has been associated with greater capillary rarefaction, inner retinal thinning, choroidal thinning, and impaired perfusion [4,38]. Kidney failure and dialysis may additionally involve impaired vascular reactivity and altered oxygenation or blood flow dynamics [11,63].

### 4.4. Diabetic Versus Non-Diabetic CKD

Diabetes is a major effect modifier in the relationship between retinal imaging phenotypes and CKD. In diabetic CKD, retinal abnormalities may reflect the combined effects of chronic hyperglycemia, hypertension, endothelial dysfunction, inflammation, diabetic retinopathy, and kidney-related microvascular injury. OCTA studies have associated lower superficial capillary density and altered choroidal measures with reduced eGFR or increased albuminuria, including among patients without clinically apparent diabetic retinopathy [42,53,55,85]. The absence of diabetic retinopathy therefore does not exclude a retinal microvascular signal associated with renal impairment. Conversely, the Singapore Prospective Study Program and Singapore Malay Eye Study cohorts showed that diabetic retinopathy was associated more consistently with albuminuric CKD than with isolated low eGFR [71]. It remains difficult to determine how much of any retinal signal is attributable specifically to kidney disease, and eGFR and albuminuria should be analyzed as complementary rather than interchangeable phenotypes.

Non-diabetic CKD provides an opportunity to examine retina–kidney associations without chronic hyperglycemic injury. A small study excluding diabetes found narrower retinal arterioles and lower AVR in 34 CKD participants than in 33 controls [86], but inconsistent eGFR equations and the small sample size limit the weight of this evidence. Other non-diabetic, hypertensive, or mixed CKD cohorts have reported capillary rarefaction, retinal neurovascular impairment, inner retinal or choroidal thinning, and altered retinal oxygenation [4,11,12,37,38,70,72,74]. Removal of diabetic confounding does not confer kidney specificity, because hypertensive and uremic vascular injury, aging, anemia, CKD etiology, and treatment may generate overlapping retinal phenotypes. Distinguishing kidney-associated retinal changes from shared cardiometabolic injury should guide comparisons across these populations. Where feasible, studies should include CKD with and without diabetes, diabetes without CKD, hypertension without CKD, and appropriately matched controls. Comparisons should characterize blood-pressure exposure, diabetes duration and control, retinopathy, cardiovascular disease, medication use, and ocular determinants alongside eGFR and albuminuria. This design would help identify retinal features associated with kidney status within comparable cardiometabolic backgrounds and distinguish them from features reflecting a broader microvascular burden. Both may be clinically informative: the former could refine kidney-related risk assessment, while the latter could support integrated cardiorenal risk enrichment.

### 4.5. Blood-Pressure Exposure, Treatment Response, and Kidney Transplantation

An important translational question is whether retinal imaging biomarkers are responsive to changes in systemic disease status. Current evidence supports biological sensitivity but does not yet establish retinal imaging as a validated treatment-response endpoint. In patients with CKD stages G3–G5, lower OCTA-derived vessel density and larger superficial flow-void areas were associated with systolic blood pressure levels and variability during the preceding year [73]. Retinal vascular caliber differences have also been reported across CKD stages in hypertensive populations [84]. These observational findings cannot determine whether improving blood-pressure control directly reverses retinal microvascular abnormalities.

Kidney transplantation provides a stronger natural model for assessing sensitivity to changes in systemic status. Prospective studies have reported increases in choroidal thickness after transplantation in parallel with improved kidney function, although not all choroidal parameters changed consistently [48]. In a series of cross-sectional and longitudinal OCT studies, retinal and choroidal thinning were associated with kidney injury and subsequent eGFR decline, while selected choroidal and retinal measurements changed following kidney transplantation or donation [4]. The parameter-specific nature of these findings argues against interpreting post-transplant changes as uniform microvascular recovery.

Changes after treatment or transplantation may reflect altered blood pressure, fluid balance, anemia, inflammation, glycemic status, or immunosuppressive exposure in addition to restored kidney function. Future studies should therefore incorporate standardized serial imaging, predefined time points, concurrent systemic measurements, appropriate control groups, and assessment of test–retest variability. Clinical translation will require evidence that imaging changes exceed measurement variability, correspond to meaningful renal or cardiovascular outcomes, and provide information beyond conventional laboratory monitoring.

## 5. Comparative Cardiorenal Contexts and Exploratory Modifiers

Cardiovascular disease, hypertension, and diabetes provide the principal comparative contexts for interpreting retinal findings in CKD because they share vascular exposures and may independently influence retinal phenotypes. Other systemic conditions are considered more briefly as potential sources of heterogeneity. Table 1 summarizes these relationships and their interpretive limitations.

### 5.1. Cardiovascular and Hypertensive Disease

Cardiovascular and hypertensive diseases share endothelial dysfunction, vascular remodeling, oxidative stress, inflammation, and impaired autoregulation with CKD [5,6]. Quantitative fundus phenotypes, including arteriolar narrowing, venular widening, tortuosity, reduced FD, and retinopathy signs, have been associated with blood-pressure exposure, hypertensive target-organ damage, and cardiovascular outcomes [5,39]. These associations are relevant to CKD studies because hypertension and cardiovascular disease can contribute to both kidney dysfunction and retinal abnormalities.

Functional measurements of vascular reactivity, blood-flow-related signals, and oxygenation may provide complementary information about systemic vascular dysfunction [11,60,61,63]. Their interpretation should account for blood pressure, cardiovascular disease, and treatment exposure. The central translational question is whether these measurements add information about kidney-related or broader cardiorenal risk beyond established clinical variables.

### 5.2. Diabetes and Cardiometabolic Disease

Diabetes is a major modifier of CKD-associated retinal phenotypes. Fundus, OCT, and OCTA studies have associated retinal vascular caliber, capillary density, and choroidal parameters with eGFR, albuminuria, and DKD [42,53,55,85,87,88]. OCTA and choroidal alterations have also been reported in participants without clinically apparent diabetic retinopathy [53,55,85]. These findings extend the detectable phenotype beyond visible retinopathy, but do not separate kidney-associated changes from the effects of diabetes itself.

Interpretation should therefore consider glycemic control, including glycated hemoglobin (HbA1c), diabetes duration, retinopathy severity, blood pressure, adiposity, medication exposure, and ocular disease. Ocular treatment history also warrants consideration: intravitreal anti-vascular endothelial growth factor (anti-VEGF) therapy and panretinal photocoagulation have been associated with changes in choroidal thickness [89,90]. Treatment type and timing relative to imaging should therefore be documented when interpreting retinal or choroidal findings in diabetic populations. Associations of retinal vascular caliber with metabolic syndrome and reported OCTA changes after metabolic interventions further illustrate the influence of the wider cardiometabolic context [91,92,93]. Existing diabetic eye-screening programs provide a practical setting for evaluating whether routinely acquired retinal information improves identification of individuals needing kidney assessment. Detailed evidence on diabetic versus non-diabetic CKD is discussed in Section 4.4, and candidate clinical pathways are considered in Section 7.

### 5.3. Other Systemic Modifiers

Liver-related, gut-related, neurodegenerative, and immune-inflammatory conditions may contribute to retinal phenotypic heterogeneity, but their relevance to CKD-specific interpretation remains uncertain. Liver–retina evidence is heterogeneous: a meta-analysis found no significant overall association between fatty liver disease and retinopathy [13]. Direct human evidence integrating microbiome profiles, quantitative retinal imaging, and CKD outcomes remains limited [14,40]. Neurodegenerative and immune-inflammatory conditions provide additional contexts for overlapping neural, vascular, and inflammatory retinal changes [6,8,44,65]. These domains are retained as potential confounders or effect modifiers rather than developed as parallel screening targets.

## 6. AI-Enabled Retinal Phenotyping and Multimodal Risk Modeling

Representative AI models are summarized in Table 3. Here, detection denotes classification of an existing kidney condition using study-defined labels, prediction estimates future onset or progression, and screening refers to a proposed workflow in which a retinal signal prompts confirmatory kidney assessment. Because task definitions, disease prevalence, imaging inputs, and validation designs differ substantially, performance estimates should not be compared directly across studies.

### 6.1. Automated Retinal Feature Extraction and Phenotype Standardization

AI can quantify predefined features, which retain biological interpretability, or learn latent image representations that may capture complex signals but are harder to separate from artifacts and confounding [5,46,94,95,96]. Reproducibility requires standardized acquisition, image quality and artifact handling, and explicit CKD labels with appropriate temporal alignment to imaging. For supervised models, labels should specify the eGFR equation, albuminuria definition, evidence of chronicity, and timing between retinal imaging and kidney measurements. Patient-level partitioning is essential to prevent leakage between eyes, visits, or repeated images [22,23].

### 6.2. AI Models for CKD Detection and Risk Prediction

Interpretation of retinal AI models requires attention to differences in kidney disease ascertainment across studies. Models detecting CKD, clinically attributed DKD, or biopsy-defined DN address distinct clinical tasks, and CKD in people with diabetes should not be assumed to represent DKD. Retinal AI studies show that fundus and OCTA images contain signals for detection of prevalent CKD or prediction of future kidney dysfunction, but external performance varies by population, setting, and device [15,16,17,18,97,98,99]. In multiethnic diabetes-screening cohorts, image-only DKD AUC decreased from 0.826 internally to 0.764 in the Singapore Epidemiology of Eye Diseases (SEED) study and 0.726 in the Singapore Macroangiopathy and Microvascular Reactivity in Type 2 Diabetes (SMART2D) study, while hybrid clinical–image models reached 0.828 and 0.761, respectively [100]; label definitions and performance still varied across cohorts. A small OCTA nomogram illustrates the limitations of interpretable feature-based models: a concordance index (C-index) of 0.678 in same-center validation, no external cohort, and no eGFR or urinary albumin excretion data [101]. Longitudinal Reti–CKD results were promising but added only modestly to eGFR–based models [15].

DeepDKD underwent broad multiethnic validation for DKD detection and was also evaluated for differentiating biopsy-defined DN from non-diabetic kidney disease (NDKD) [19]. Its smaller biopsy cohorts and attenuated external performance support use as a potential triage aid rather than a substitute for nephrology assessment or biopsy.

Biopsy-linked systems have extended retinal AI to pathological classification and nephrology triage. KIDS explored CKD screening, pathological classification, and prognosis, whereas Trans-MUF differentiated DN from non-diabetic renal disease (NDRD) using retinal and clinical information [102,103]. Their interpretation remains task-specific: selected biopsy populations, small external cohorts, and differences between image-level and participant-level performance affect applicability. Detailed model characteristics and validation results are summarized in Table 3.

The evidence now includes external validation and prospective proof-of-concept evaluation, but these should be distinguished from studies demonstrating benefit from model-guided care [16,17,18,19,102,103]. The key translational questions are whether performance transfers across settings, whether retinal information adds value beyond clinical assessment, and whether its use improves testing or management decisions.

### 6.3. Multimodal Risk Modeling Across Retinal and Systemic Biomarkers

Clinically meaningful incremental value beyond conventional assessment remains limited. Reti–CKD improved the C-index by 0.020–0.024 over eGFR-based comparators in cohorts that excluded participants with baseline eGFR < 90 mL/min/1.73 m^2^ or proteinuria [15]. This setting differs from the Kidney Failure Risk Equation (KFRE), which uses age, sex, eGFR, and UACR to estimate kidney-failure risk in people with established CKD [104]. These findings support evaluating retinal models for risk enrichment or opportunistic case finding when recent kidney laboratory results are unavailable. Retinal-plus-urine or image–clinical models have not consistently outperformed simple clinical comparators in external cohorts [16,97].

Comparators should reflect the intended decision point. When recent kidney tests are unavailable, retinal models should be evaluated against clinical risk assessment and relevant targeted or universal eGFR/UACR testing strategies. When laboratory results are available, retinal features should demonstrate added value beyond kidney and cardiometabolic variables. Improvements over image-only models do not establish the incremental contribution of retinal imaging; discrimination gains should be considered alongside calibration and decision-analytic net benefit [26,105].

**Table 3:** **Representative retinal AI models for kidney disease assessment.**

Study/Model	Clinical Task	Imaging Input	Development/Validation Cohorts	Discrimination and Incremental Value	External Validation/Domain Shift	Calibration, Utility, and Key Limitations
Sabanayagam et al., 2020; image and hybrid DLA [16]	Prevalent CKD detection: eGFR < 60 mL/min/1.73 m^2^	Bilateral macula-centered fundus photographs; image-only or combined with conventional risk factors	SEED: development *n* = 5188; internal validation *n* = 1297. External testing: SP2 *n* = 3735; BES *n* = 1538	AUCs in SEED/SP2/BES: image-only DLA, 0.911/0.733/0.835; RF model, 0.916/0.829/0.887; hybrid DLA, 0.938/0.810/0.858	Independent external testing in SP2 and BES; marked attenuation of image-only performance in SP2	Hybrid DLA improved on image-only discrimination but did not outperform RF externally, highlighting the importance of clinical comparators.
Zhang et al., 2021; image-only and combined models [17]	Prevalent CKD detection, eGFR estimation, and exploratory incident-CKD prediction	Color fundus photographs, with/without metadata; prospective smartphone imaging	Development: 43,156 participants/86,312 images. External cohorts: *n* = 8059 and 3081. Overall: 57,672 participants/115,344 images	Image AUC: 0.918 internally, 0.885 in external test 1, and 0.870 in the smartphone test; combined AUC: 0.930, 0.898, and 0.897; eGFR MAE: 11.1–13.4 mL/min/1.73 m^2^	Geographically distinct Chinese cohorts and prospective smartphone testing supported cross-setting/device feasibility; ethnic and health-system diversity remained limited.	Metadata gains were modest; kidney specificity and prospective clinical utility remain unestablished
Joo et al., 2023; Reti–CKD score [15]	Incident-CKD prediction in people with preserved kidney function	Retinal deep-learning score combined with clinical variables in a Cox model	Retinal DLA development: Korean screening cohort, *n* = 79,108. Longitudinal evaluation: UK Biobank, *n* = 30,477; Korean Diabetic Cohort, *n* = 5014	Highest versus lowest score quartile: HR 3.68 in UK Biobank and 9.36 in the Korean cohort. C-index gains over the study’s eGFR-CKD score: 0.020/0.024, respectively	Cox score fitted in UK Biobank and externally tested in the Korean Diabetic Cohort, distinct from Korean DLA development. Participants with baseline eGFR < 90 mL/min/1.73 m^2^ or proteinuria were excluded.	Longitudinal prognostic evidence, with modest incremental concordance; clinical thresholds and applicability to established CKD remain unresolved
Zhao et al., 2024; UWF–CKDS [18]	Prevalent CKD detection within a screening workflow	200° UWF images, derived AVR/FD/tortuosity, and selected medical history; comparator: central 50° crops	Internal: 7781 participants/23,313 images. Prospective multicenter test: 1352 participants/3226 images across 23 Chinese tertiary hospitals	UWF-CKDS AUC: 0.86 internally and 0.81 in multicenter testing; central-region comparator: 0.82/0.77	Prospective multicenter testing within China; UWF-specific acquisition, tertiary ophthalmic sampling, and absent non-Chinese validation limit transportability	UWF outperformed central crops; added value beyond clinical-only assessment remains unresolved. Equipment and workflow costs may limit implementation.
Bhak et al., 2025; eGFR–MMDL [97]	Detection of eGFR < 60 mL/min/1.73 m^2^	Bilateral fundus images plus age, sex, and urine dipstick measurements	Development/internal testing: CHA Bundang Medical Center, *n* = 65,082. External validation: Severance Checkup Health Promotion Center, *n* = 58,284	MMDL versus image-only AUC: 0.94 versus 0.90 internally; 0.88 versus 0.77 externally. Age/sex/urine-dipstick logistic model performed comparably to MMDL externally	Independent Korean center and different cameras; lower performance in adults ≥65 years; no non-Korean validation	Supports retinal–urine integration, but limited gain over the non-image comparator warrants cost and workflow evaluation
Meng et al., 2025; DeepDKD [19]	DKD detection; differentiation of isolated DN from NDKD	Fundus images; metadata-based comparators	DKD task: development *n* = 121,578; external validation *n* = 65,406 across 10 multiethnic cohorts. Biopsy task: development *n* = 267; external validation *n* = 244 across 3 cohorts	DKD AUC: 0.842 internally, 0.791–0.826 externally; DN versus NDKD AUC: 0.906 internally, 0.733–0.844 externally. Prospective proof-of-concept DKD sensitivity: 89.8% versus 66.3% for metadata alone (*p* < 0.0001)	Multiethnic, multicountry external validation with performance attenuation; biopsy task used smaller, selected cohorts	Prospective screening proof of concept included 3-month follow-up; management/outcome benefits require evaluation. Biopsy-task findings support triage research, not replacement of indicated biopsy.
Wu et al., 2025; KIDS [103]	CKD detection; biopsy-defined pathological classification; severe glomerulosclerosis detection; exploratory progression prediction	Bilateral 45° fundus photographs; image-only screening and retinal–clinical pathological models	Overall: 6773 participants/13,144 images across 9 hospitals. Screening: development/internal testing *n* = 3965; external testing *n* = 743. Pathological models: 2139 biopsy-confirmed patients	Screening AUC: 0.959 internally/0.858 externally; external hybrid pathological AUCs: 0.867–0.936. Real-world AUCs of 0.790–0.932 included clinical-only testing without retinal images. Prognostic C-index: 0.804	Prospective multicenter/cross-device testing; predominantly Chinese retinal cohorts. Some multiethnic datasets lacked retinal images. Selected cases/controls risked spectrum bias; prognostic model lacked external validation	Calibration assessed for pathological models; exception noted for hybrid ANS. Images improved NRI/IDI, but AUC gains over clinical-only models were nonsignificant after Bonferroni correction. Clinical triage utility requires evaluation
Dong et al., 2025; Trans–MUF [102]	Biopsy-defined DN versus NDRD in adults with type 2 diabetes and CKD	Fundus photographs, automated lesion maps, and five clinical variables: diabetes history, systolic BP, eGFR, hemoglobin, and HbA1c	Retrospective development/internal validation: 246 patients/934 images. Prospective same-center temporal validation: 41 patients. External validation: 28 patients/55 images from 5 Chinese hospitals	Internal/prospective/external AUCs: image-wise, 0.980/0.989/0.932; patient-wise, approximately 0.973/0.979/0.876	Prospective testing was same-center temporal validation. Multicenter/cross-device external testing showed lower patient-level performance; the external cohort was small and exclusively Chinese	Ablation, explainability, reader evaluation, and DCA supported further biopsy-triage research; routine biopsy avoidance remains unsupported
Tshedron and Li, 2025; OCTA nomogram [101]	Source-defined DN classification during DR screening	OCTA DCP parafoveal density, FD-300, age, sex, and axial length	Retrospective single-center dataset: 464 eyes; random 7:3 split into training (324 eyes) and internal validation (140 eyes)	Training/internal-validation C-indices: 0.728/0.678; bootstrap estimates: 0.747/0.681	No independent external validation; eGFR and urinary albumin data were not collected; DN criteria insufficiently detailed	Calibration curves and DCA provided internal evidence only. Proof of concept requiring external validation and prospective clinical-impact evaluation

**Note:** Models were selected as representative examples of kidney-related tasks and validation settings; see Supporting Information S1, Section 3 and Section 4. Performance estimates are not directly comparable across differing tasks, populations, disease definitions, imaging inputs, and validation designs. Same-institution hold-out sets represent internal validation; incremental value depends on the comparator. Calibration, decision-analytic evidence, and prospective testing do not alone establish benefit from model-guided care. Disease labels follow the source studies: DN denotes biopsy-confirmed diabetic nephropathy where documented; otherwise, the source label is retained with diagnostic limitations stated. NDKD/NDRD terminology follows the source, with biopsy confirmation specified where applicable. **Abbreviations:** AUC, area under the receiver operating characteristic curve; AVR, arteriolar-to-venular ratio; BES, Beijing Eye Study; BP, blood pressure; C-index, concordance index; CKD, chronic kidney disease; DCA, decision-curve analysis; DCP, deep capillary plexus; DKD, diabetic kidney disease; DLA, deep learning algorithm; DN, diabetic nephropathy; eGFR, estimated glomerular filtration rate; FD, fractal dimension; FD-300, foveal capillary density in the 300 μm annulus surrounding the foveal avascular zone; HbA1c, glycated hemoglobin; HR, hazard ratio; MAE, mean absolute error; MMDL, multimodal deep learning; NDKD, non-diabetic kidney disease; NDRD, non-diabetic renal disease; OCTA, optical coherence tomography angiography; RF, risk-factor model; SEED, Singapore Epidemiology of Eye Diseases; SP2, Singapore Prospective Study Program; UACR, urinary albumin-to-creatinine ratio; UWF, ultra-widefield imaging; ANS, arterionephrosclerosis; IDI, integrated discrimination improvement; NRI, net reclassification improvement; AI, artificial intelligence; DR, diabetic retinopathy.

### 6.4. Generalizability, Calibration, and Subgroup Performance

The external performance attenuation observed in retinal CKD models makes transportability a central interpretive issue [16,17,18,19,97]. Evaluation should distinguish independent external cohorts from same-center hold-out testing and document differences in population, imaging device, field of view, and kidney-outcome definitions. In particular, differences in diabetes and retinopathy status, CKD severity, and eGFR or albuminuria ascertainment may alter both the retinal signal and the clinical task.

Discrimination should be considered alongside calibration and performance at thresholds relevant to confirmatory kidney testing [24]. Subgroup assessment should examine clinically consequential errors and ungradable-image rates, as well as overall accuracy, because pooled performance may conceal differences across age, ethnicity, diabetes status, ocular comorbidity, and devices [21]. These assessments determine whether a model can support the intended CKD-testing pathway in a new setting. Supporting frameworks include the Transparent Reporting of a multivariable prediction model for Individual Prognosis Or Diagnosis plus artificial intelligence (TRIPOD+AI) reporting guideline and the Prediction model Risk Of Bias ASsessment Tool plus artificial intelligence (PROBAST+AI) appraisal tool [22,23].

### 6.5. Explainability, Decision Curve Analysis, and Clinical Utility

Explainability is relevant when interpreting whether retinal models rely on measurable vascular or structural features, ocular disease, or shared cardiometabolic signals. Linking model outputs to quantified retinal phenotypes can strengthen biological interpretation, whereas plausible heatmaps alone do not establish kidney specificity or causality [20,106]. Clinical usefulness nevertheless depends on the decision supported by the model, rather than the plausibility of its visual explanation.

For a proposed action such as confirmatory eGFR/UACR testing, decision-curve analysis should compare retinal-guided assessment with relevant clinical-only, test-all, test-none, and usual-care strategies across clinically plausible thresholds [26,105]. This provides decision-analytic evidence, which should remain distinct from prospective model validation and clinical-impact evaluation. Prospective studies should determine whether retinal guidance improves appropriate testing and referral, shortens time to kidney assessment, and provides benefits that justify false alerts, workload, and costs [25,26]. The proposed clinical framework is discussed in Section 7.

## 7. Potential Clinical Applications and Requirements for Prospective Evaluation

### 7.1. Opportunistic Screening in Ophthalmology Settings

Translation is most plausible where retinal images are already acquired, particularly in routine eye care and diabetic screening [8]. Conventional FP provides the most scalable starting point for prospective evaluation of opportunistic CKD risk enrichment. UWF imaging may extend retinal coverage where available, while quantitative OCT/OCTA assessment offers a complementary research approach. Quantitative features or AI-derived risk could flag patients with no recent kidney assessment for confirmatory testing [16,17,19]. Diabetic eye screening is the most practical initial setting because imaging infrastructure, repeated attendance, and a high risk of kidney disease coexist. Retinal outputs should trigger a defined action—such as eGFR/UACR testing or primary-care review—rather than an unexplained systemic risk score; programs must track image quality, referral completion, and patient communication [107,108].

### 7.2. CKD Risk Enrichment Rather than Stand-Alone Diagnosis

Retinal imaging should be positioned as a risk-enrichment tool, not a stand-alone diagnostic test. CKD diagnosis and staging remain based on eGFR, UACR, and chronicity [2,3]. Because retinal vascular, structural, perfusion, and AI-derived features are influenced by systemic, ocular, and technical factors, an elevated signal should prompt confirmatory assessment rather than be used to assign a CKD label. Clinical value must be judged against existing risk-based or universal testing strategies, not image interpretation alone [5,6,9,19,39,99].

### 7.3. Proposed Framework for Prospective Evaluation

**Figure 4:**
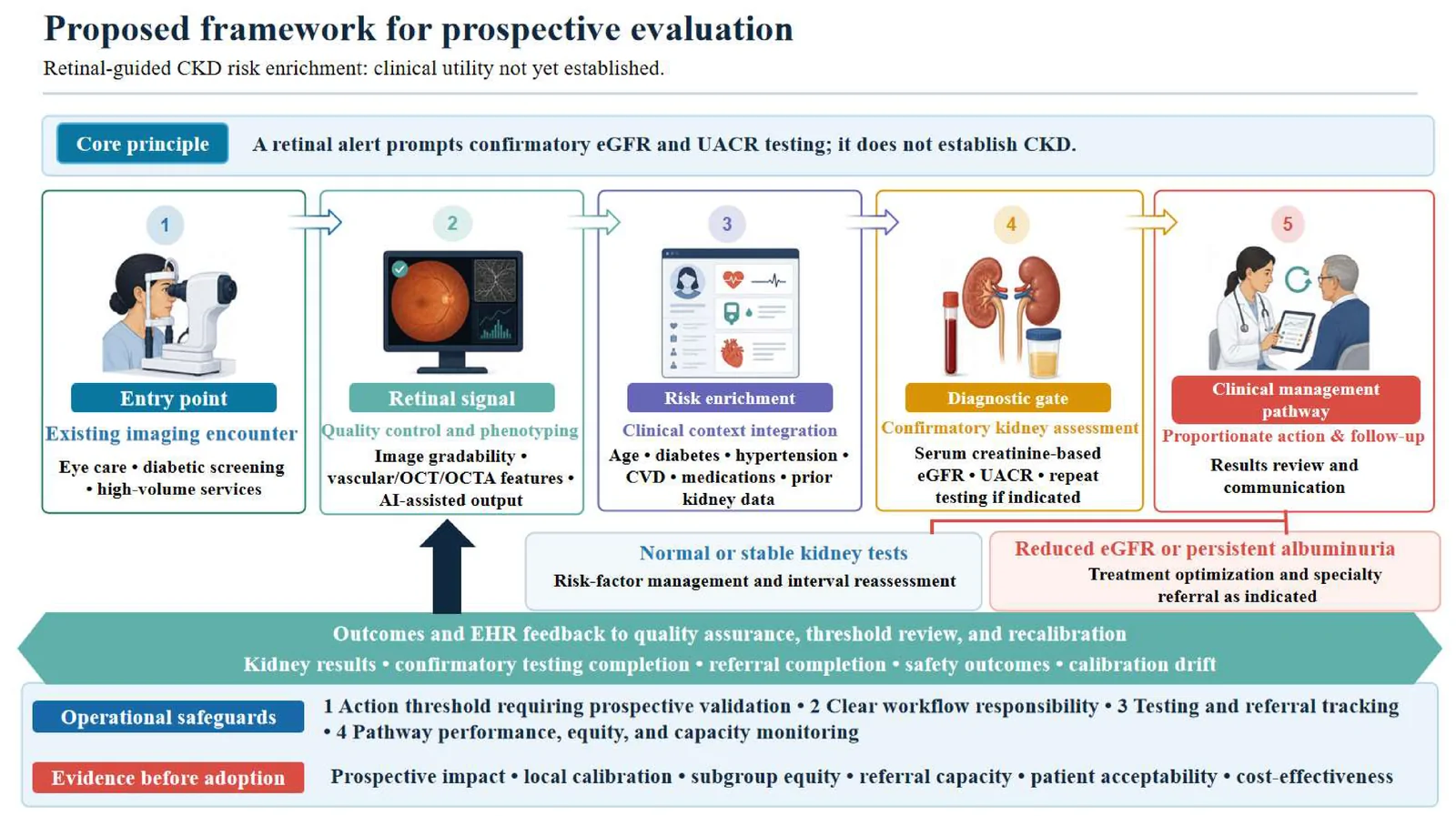
**Proposed framework for prospective evaluation of retinal imaging-guided CKD risk enrichment.** The schematic illustrates how retinal signals could be integrated with clinical context to prompt confirmatory eGFR and UACR testing, followed by results communication and management according to established clinical guidance. Feedback could support quality assurance and model monitoring. The retinal-guided framework has not been established as a clinically validated pathway: actionable retinal thresholds, improved patient outcomes, cost-effectiveness, and generalizable workflow performance require prospective evaluation. The arrows indicate proposed information and care processes, not demonstrated clinical benefit. AI, artificial intelligence; CKD, chronic kidney disease; CVD, cardiovascular disease; eGFR, estimated glomerular filtration rate; EHR, electronic health record; OCT, optical coherence tomography; OCTA, optical coherence tomography angiography; UACR, urinary albumin-to-creatinine ratio.

Figure 4 presents a proposed framework for prospective evaluation, rather than a clinically validated pathway. It links an existing retinal imaging encounter, quality control and phenotyping, integration with clinical context, confirmatory kidney assessment, and proportionate follow-up. Within this proposed framework, retinal findings would be interpreted alongside age, diabetes and hypertension status, cardiovascular history, medication exposure, and previous kidney results. A retinal signal meeting a prospectively specified study threshold would prompt consideration of serum creatinine-based eGFR and UACR testing, rather than establish a CKD diagnosis [2,3].

Once kidney abnormalities are identified, their evaluation and management should follow established clinical pathways. Normal kidney tests may lead to routine risk–factor management and interval reassessment. Reduced eGFR, persistent albuminuria, a sustained clinically meaningful decline in eGFR on serial testing, resistant hypertension, or suspected DKD should prompt management according to local guidance, ranging from primary-care optimization and endocrinology consultation to nephrology referral. Kidney results, referral completion, safety events, and subsequent outcomes should feed back into the linked imaging and electronic health record (EHR) system for quality assurance, calibration monitoring, governed threshold review, and model recalibration. Prospective implementation studies should test whether this closed loop improves case finding and net clinical benefit rather than merely increasing referrals [25,26,105].

The framework does not specify validated retinal action thresholds, and improved patient outcomes, cost-effectiveness, and generalizable workflow performance have not been established for this proposed pathway. Prospective studies should define the intended population and action thresholds, assign responsibility for communication and confirmatory testing, and compare the retinal-guided strategy with relevant clinical or testing alternatives [25,26,105,109]. Evaluation should include testing and referral completion, false-alert burden, subgroup equity, service capacity, patient outcomes, and costs [21,25,110].

### 7.4. Integration with Systemic Biomarkers and Electronic Health Records

Retinal phenotypes may reflect accumulated microvascular, neuroretinal, and choroidal injury, whereas eGFR, UACR, and their longitudinal trajectories establish kidney status, and blood pressure, HbA1c, and medication history inform clinical actionability. Some multimodal models improve performance or stability, but gains remain task- and population-dependent [16,17,97]. EHR integration should serve the decision rather than simply add predictors. The system should identify missing or outdated kidney tests, display the retinal signal alongside prior eGFR/UACR results and relevant comorbidities, document the action taken, and capture downstream outcomes.

### 7.5. Implementation in Resource-Limited and High-Volume Settings

High-volume and resource-limited services may benefit from retinal risk enrichment, but implementation should begin with the simplest sustainable pathway. Non-mydriatic or handheld FP, automated image-quality assessment, and targeted eGFR/UACR testing are more scalable than deploying specialist OCTA or functional imaging solely for CKD screening. Teleophthalmology and real-world diabetic retinopathy screening programs demonstrate that non-specialist acquisition and automated triage can extend reach, while unreadable images, staff training, referral completion, and downstream capacity remain decisive constraints [107,108].

Local validation must address camera and illumination differences, disease prevalence, rates of ungradable images, subgroup performance, laboratory and referral capacity, acceptability, and workload [21,25,107]. A system that identifies more high-risk patients but exceeds confirmatory testing or referral capacity may provide little net benefit.

### 7.6. Candidate Clinical Scenarios for Prospective Evaluation

Table 4 summarizes candidate clinical scenarios and their principal evidence gaps. Diabetic eye screening is a priority setting for prospective evaluation because retinal images are routinely acquired and externally validated CKD/DKD models are available [16,17,19]. In established CKD, dialysis, and post-transplant care, quantitative and functional imaging offers a complementary research setting for longitudinal phenotyping [4,11,61,63]. Broad screening of average-risk asymptomatic populations is not currently justified.

### 7.7. Evidence Needed Before Routine Adoption

Routine adoption requires a linked evidence chain: analytical validity (acquisition reliability, repeatability, failure detection, and cross-device performance); clinical validity (external validation, calibration, subgroup performance, and incremental value beyond routine kidney and cardiometabolic variables); and clinical utility. Workflow-embedded studies should determine whether retinal guidance improves appropriate eGFR/UACR testing, referral, and risk-factor management and provides net clinical benefit without disproportionate false alerts, anxiety, inequity, or workload [25,26,105]. Pragmatic and health-economic evaluation should precede broad adoption.

## 8. Barriers and Methodological Challenges

### 8.1. Imaging Standardization and Inter-Device Reproducibility

Technical variation in camera characteristics, field of view, illumination, scan protocol, segmentation, artifact correction, and software can obscure subtle biological differences. Even acceptable within-device repeatability does not ensure cross-platform agreement: seven OCTA devices produced non-interchangeable measurements of vessel density, FD, and FAZ [111]. Device-specific thresholds should therefore not be transferred across platforms without harmonization and independent validation. Studies should report device, acquisition, and software details, quality thresholds, exclusions, segmentation review and correction, and harmonization procedures. For longitudinal studies, device- and protocol-specific intraclass correlation coefficients, coefficients of variation, and minimal detectable change should be estimated before an observed change is interpreted as biological progression or treatment response [58,59]. Studies using bilateral images should prespecify eye-selection rules or account for within-participant correlation, and should clearly distinguish eye-level from participant-level outcomes.

### 8.2. Population Heterogeneity and Confounding

Retinal phenotypes in CKD are composite signals shaped by kidney status, systemic vascular exposure, ocular disease, and technical factors. Adjustment quality is uneven across the literature: age and sex are commonly included, whereas contemporaneous blood pressure and antihypertensive treatment, diabetes duration, HbA1c and diabetic retinopathy severity, hemoglobin or anemia, albuminuria and CKD etiology, cardiovascular risk, and ocular determinants such as axial length, refractive error, ocular comorbidities, and image quality are handled inconsistently [4,5,6,9,10,39,71,73,75,80,83]. Consequently, fully adjusted, time-aligned longitudinal analyses should carry greater interpretive weight than minimally adjusted cross-sectional comparisons; residual confounding nevertheless continues to preclude kidney-specific attribution.

Outcome definitions are also inconsistent. CKD may be defined using a single eGFR value, repeated measurements, albuminuria, clinical coding, or combinations of these criteria, while DKD, hypertensive nephropathy, glomerular disease, kidney failure, and transplant populations are often pooled [2,3]. Several retinal studies classified eGFR 60–89 mL/min/1.73 m^2^ as mild CKD without documenting chronicity or another marker of kidney damage, and others defined incident CKD using a single follow-up eGFR < 60 mL/min/1.73 m^2^. This heterogeneity can inflate or dilute associations and makes subgroup estimates difficult to transport. Analyses should prespecify kidney definitions, distinguish major etiologic and treatment groups when feasible, and evaluate interactions rather than relying only on covariate adjustment. For AI models, subgroup discrimination, calibration, missingness, and rates of image-quality-related failure should be reported because an acceptable pooled AUC can conceal clinically important underperformance [21,22].

The evidence base is geographically concentrated, with many retinal CKD and AI studies derived from East and Southeast Asian cohorts. Transportability to populations in Africa, Latin America, and the Middle East, as well as to Indigenous and other under-represented populations, remains uncertain because retinal pigmentation, CKD etiology, cardiometabolic burden, imaging devices, disease prevalence, and healthcare context may affect both measured phenotypes and model calibration [15,16,17,18,19,97].

### 8.3. Organ Specificity and Causal Interpretation

A central interpretive task is to distinguish retinal features associated with kidney status from those reflecting shared cardiometabolic microvascular burden. Vascular geometry, OCT/OCTA measures, and neuroretinal or choroidal structure may reflect overlapping influences of CKD, diabetes, hypertension, aging, ocular disease, and treatment [5,6,9]. Statistical adjustment is important, but clinically informative comparator groups provide an additional means of examining whether associations differ across kidney and cardiometabolic phenotypes. Organ specificity and clinical usefulness are distinct questions: a retinal feature need not be kidney-specific to contribute to risk assessment, provided that its incremental value is demonstrated for a defined clinical task.

Most evidence is cross-sectional and cannot establish whether retinal changes precede kidney dysfunction, develop in parallel, or reflect shared exposures. Longitudinal studies remain vulnerable to residual confounding, competing risks, treatment effects, measurement error, and reverse causation through the consequences or treatment of kidney disease, including uremia, anemia, fluid shifts, dialysis, and immunosuppression [4,48,73]. Mechanistic claims should therefore not exceed study design; current evidence supports complementary risk phenotyping rather than a causal or CKD-specific signature. Emerging causal-inference studies may help clarify directionality. A 2026 bidirectional two-sample Mendelian randomization analysis suggested associations in both kidney-to-retina and retina-to-kidney directions; however, restriction to a specific ancestry, heterogeneous DKD phenotypes, multiple testing, and instrument selection assumptions make these findings hypothesis-generating rather than definitive evidence of causality [112].

### 8.4. AI Deployment, Governance, and Workflow Integration

Deployment requires a defined target population, specified eligible image types and compatible devices, action thresholds, failure handling, human oversight, responsibility for confirmatory testing or referral, and post-deployment monitoring. Governance should cover data provenance, cybersecurity, privacy, auditability, version control, calibration drift, subgroup performance, human–AI interaction, alert burden, and unintended consequences [21,25]. Governance and workflow integration should therefore be treated as components of clinical performance rather than as administrative steps undertaken after model validation.

## 9. Future Roadmap

**Figure 5:**
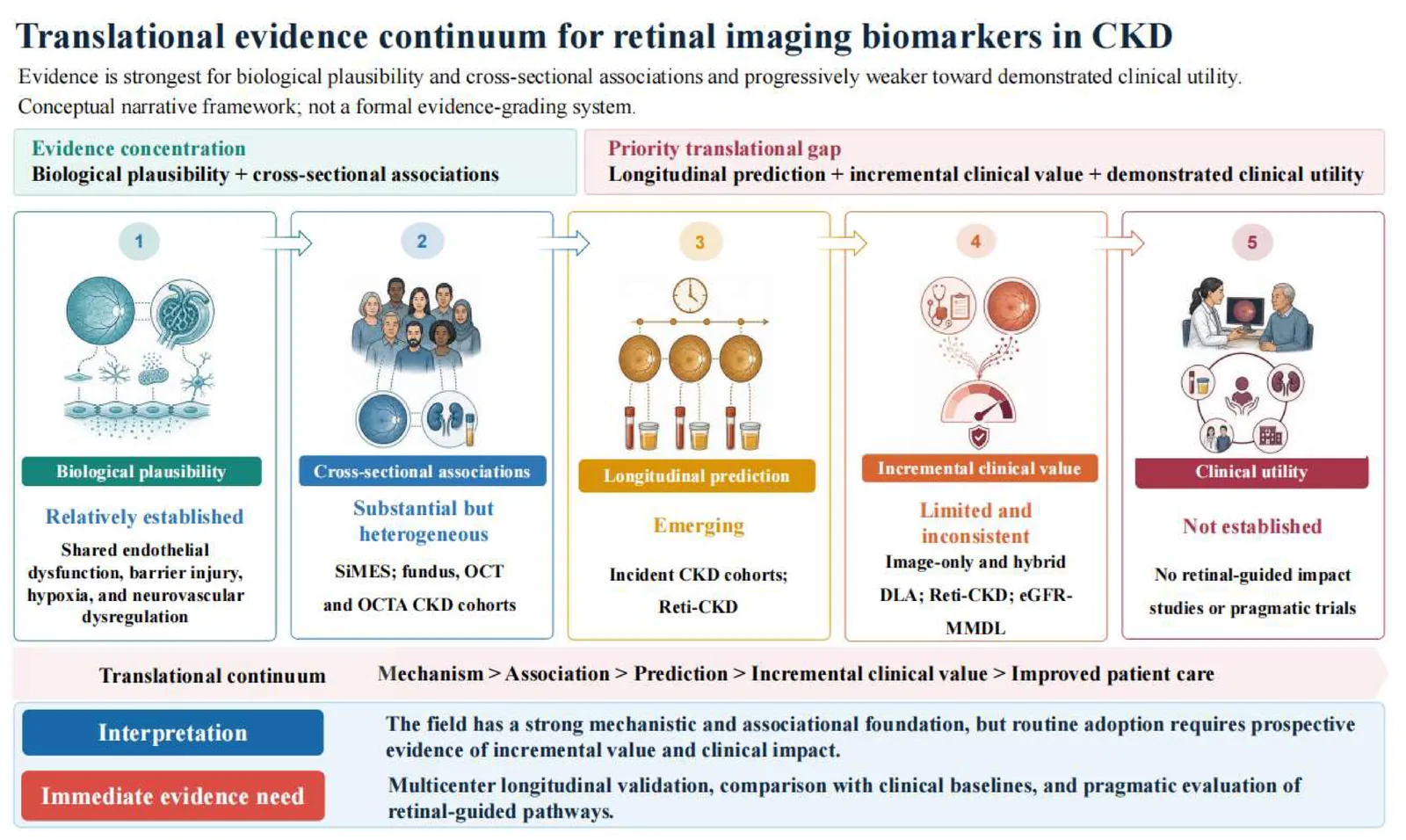
**Translational evidence continuum for retinal imaging biomarkers in CKD.** This conceptual narrative framework describes the maturity of the translational evidence chain. It is not a formal Grading of Recommendations Assessment, Development and Evaluation (GRADE) assessment. Biological plausibility and cross-sectional association do not establish incremental clinical value. Incremental value requires comparison with a prespecified clinical baseline, alongside calibration, subgroup performance, and decision-analytic benefit; routine clinical utility remains unestablished. CKD, chronic kidney disease; DLA, deep learning algorithm; eGFR, estimated glomerular filtration rate; MMDL, multimodal deep learning; OCT, optical coherence tomography; OCTA, optical coherence tomography angiography; SiMES, Singapore Malay Eye Study.

Figure 5 summarizes the translational evidence continuum: biological plausibility and cross-sectional associations are comparatively well developed, whereas longitudinal prediction, incremental clinical value, and clinical utility remain progressively less established [5,16,99].

### 9.1. Multicenter Prospective Cohorts with Standardized Imaging Protocols

The immediate priority is multicenter prospective cohorts designed to distinguish kidney-associated retinal signals from shared cardiometabolic microvascular burden. Recruitment should, where feasible, incorporate the comparator groups outlined in Section 4.4, with time-aligned retinal imaging, eGFR, UACR, treatment exposures, and clinical outcomes. Prespecified analyses should assess within-group associations and interactions by diabetes and hypertension status, alongside standardized acquisition, quality control, device harmonization, and test–retest reliability [4,9,35,46,58,59,111]. Nested AI analyses should lock model specifications before outcome evaluation and separate development and validation by site, device, geography, or time. Such cohorts can support longitudinal biomarker validation, treatment-response assessment, and subsequent pragmatic trials, moving the field beyond isolated cross-sectional analyses.

### 9.2. Multi-Organ Biomarker Panels and Longitudinal Assessment

Biomarker development should use a tiered strategy. A deployable core panel could combine retinal morphology or perfusion features with eGFR, UACR, blood pressure, diabetes status, HbA1c, medication exposure, and cardiovascular risk. A discovery tier could then add circulating or urinary markers of endothelial injury, inflammation, oxidative stress, uremic exposure, extracellular vesicle signaling, and selected omics data. This tiered structure would test whether retinal phenotypes identify a cross-organ microvascular state while preserving a pathway toward routine implementation [6,34]. The goal is the smallest panel that improves a prespecified clinical decision. Nested-model or ablation analyses should quantify retinal, laboratory, and omics contributions against a parsimonious clinical baseline.

Longitudinal studies should time-align repeated retinal imaging with eGFR and UACR trajectories, treatments and medications, fluid status, hemoglobin, blood pressure, omics measurements where available, and clinical outcomes. Imaging intervals should be prespecified, and the provenance and timing of laboratory, imaging, omics, and outcome data should be preserved in linked research datasets or EHRs.

### 9.3. Pragmatic Trials and Real-World Implementation

Once prospective clinical validity is established, evaluation should progress from silent-mode workflow testing to pilot impact studies and pragmatic or cluster-randomized trials. Recent ophthalmology-specific literature describes AI applications in trial recruitment, patient monitoring, and outcome assessment [113]. This provides complementary context for planning retinal CKD studies, while the clinical benefit of a retinal-guided kidney-testing strategy requires evaluation within its intended care setting. Outcomes should cover testing completion, newly detected reduced eGFR or albuminuria, referral appropriateness, time to review, workload, resource use, patient outcomes, and equity; decision-curve and health-economic analyses should test whether benefits outweigh the harms and costs associated with false alerts and over-referral [26,105].

### 9.4. Ophthalmology as a Partner in Systemic Microvascular Risk Assessment

Ophthalmology can provide an entry point for systemic risk recognition through existing eye-care and diabetic-screening pathways [8]. A sustainable shared-care model requires structured retinal reports, EHR interoperability, agreed testing and referral criteria, clear responsibility for patient communication, and feedback across eye care, primary care, endocrinology, cardiology, and nephrology. Training should prepare eye-care professionals to recognize and communicate systemic risk without transferring responsibility for kidney diagnosis or management into ophthalmology.

## 10. Conclusions

Retinal imaging provides noninvasive access to systemic microvascular and neurovascular phenotypes. CKD is a useful translational reference because eGFR, UACR, disease severity, and treatment transitions offer clinical anchors for evaluating retinal measurements. The same signals may arise from shared cardiovascular, metabolic, inflammatory, ocular, and aging-related processes [2,3,4,5,6,8,9].

Retinal imaging offers a promising adjunctive approach to systemic microvascular risk enrichment, particularly when routinely acquired images can identify individuals needing kidney assessment. Prospective studies should establish the incremental clinical utility of retinal-guided strategies beyond existing clinical approaches by assessing testing, referral, and patient outcomes, while addressing image quality, device compatibility, actionable thresholds, responsibility for confirmatory testing, and downstream service capacity [2,3,25,26,99]. Ophthalmology can contribute to this evaluation through coordinated care with primary care and relevant specialties.

## Figures and Tables

**Table 1:** **Comparative biological features, characteristics of the available evidence, and interpretive roles of multi-organ–eye relationships.**

Relationship/Modifier	Principal Systemic Drivers	Shared Mechanisms	Candidate Retinal Phenotypes	Characteristics of the Available Evidence	Major Interpretive Limitations
Retina–kidney relationship	Reduced kidney function; uremic milieu; hypertension; diabetes; anemia; RAAS and volume dysregulation	Endothelial and barrier dysfunction; oxidative stress/inflammation; capillary rarefaction and hypoxia	Vascular geometry; OCTA density/FAZ; retinal/choroidal thickness; oxygenation, flow, and reactivity	Repeated cross-sectional associations; fewer longitudinal and treatment-context studies [4,9,12,35]	Heterogeneous CKD etiologies; confounding by diabetes, hypertension, age, ocular disease, and treatment; no validated kidney-specific thresholds
Cardiovascular/hypertensive context	Hypertension; arterial stiffness; atherosclerotic and cerebrovascular burden	Hemodynamic stress; endothelial dysfunction; vascular remodeling; impaired autoregulation and vasodilation	Arteriolar narrowing/venular widening; AVR, FD, and tortuosity; retinopathy; perfusion and vascular reactivity	Extensive observational evidence, including prospective studies [5,39]	Hypertension duration and treatment; overlap with diabetes and CKD; limited cardiovascular specificity
Diabetes/ cardiometabolic context	Hyperglycemia; insulin resistance; obesity; dyslipidemia; metabolic syndrome	AGE-related and oxidative injury; pericyte loss; barrier and NVU dysfunction; inflammation	DR lesions; capillary nonperfusion; density/FAZ; neuroretinal thickness; oxygenation and reactivity	Extensive diabetic retinal disease evidence; less evidence for systemic and kidney risk prediction [10,42]	Diabetes duration, glycemic control, retinopathy severity, treatment, and hypertension may dominate retinal findings
Liver-related modifier *	MASLD; insulin resistance; obesity; dyslipidemia; hepatic inflammation and fibrosis	Metabolic dysregulation; inflammation; oxidative stress; endothelial dysfunction	Vascular lesions and geometry; exploratory OCTA and choroidal measures	Limited and heterogeneous human evidence [13,43]	Diabetes and obesity confounding; variable liver-disease definitions; predominantly cross-sectional evidence; limited liver specificity
Gut-related modifier *	Dysbiosis; intestinal barrier dysfunction; diet/medications; microbial metabolites	Immune activation; systemic and neuroinflammatory signaling; altered microbial metabolites	No established phenotype; exploratory perfusion, structural, and inflammatory measures	Mainly mechanistic and ocular-disease evidence; limited direct CKD–retinal evidence [14,40]	Geographic, dietary, medication, and microbiome variation; reverse causality; limited longitudinal evidence
Neurodegenerative and immune-inflammatory context	Neurodegeneration; autoimmunity; systemic inflammatory disease; immune dysregulation	Neurovascular uncoupling; microglial activation; endothelial/barrier injury; neuronal loss	RNFL/ganglion-cell measures; OCTA perfusion; vascular reactivity; disease-specific inflammatory findings	Heterogeneous, disease-specific evidence; exploratory for systemic microvascular assessment [8,44]	Disease-specific retinal changes; potentially primary ocular involvement; limited organ specificity and causal evidence

* Exploratory modifier; current evidence does not support its use as an independent screening target. **Note:** Evidence descriptions reflect the authors’ qualitative narrative interpretation, considering study design, consistency, and longitudinal or validation evidence where applicable. They were not assigned using prespecified criteria or a formal grading procedure and do not establish clinical readiness. None of the listed retinal phenotypes has been validated as an organ-specific diagnostic biomarker. **Abbreviations:** AGE, advanced glycation end product; AVR, arteriolar-to-venular ratio; CKD, chronic kidney disease; FAZ, foveal avascular zone; FD, fractal dimension; MASLD, metabolic dysfunction-associated steatotic liver disease; NVU, neurovascular unit; OCTA, optical coherence tomography angiography; RAAS, renin–angiotensin–aldosterone system; RNFL, retinal nerve fiber layer; DR, diabetic retinopathy; OCT, optical coherence tomography.

**Table 4:** **Candidate clinical scenarios for prospective evaluation of retinal imaging-based CKD risk enrichment.**

Clinical Scenario	Target Population/Entry Point	Imaging and Analysis	Action After an Elevated Signal	Current Translational Position	Principal Evidence Gaps or Safeguards
**Diabetic eye screening**	People with diabetes undergoing routine retinal screening; no recent kidney assessment	Existing fundus or UWF images; quantitative phenotypes or an appropriately validated AI-derived risk output	Review existing eGFR and UACR results or obtain serum creatinine-based eGFR and UACR testing; primary-care or diabetes–team review	Most ready for prospective evaluation	CKD-specific impact, actionable thresholds, cost–effectiveness, and referral completion
**Targeted use in routine ophthalmology**	Older adults or patients with hypertension, CVD, obesity, or metabolic disease	FP with OCT/OCTA when already clinically acquired; EHR-supported interpretation	Arrange confirmatory kidney testing and optimize cardiometabolic risk; refer according to conventional criteria	Candidate for targeted prospective evaluation	Limited organ specificity, confounding by ocular disease, and incremental value beyond clinical risk factors
**High-volume or resource-limited triage**	Diabetes or hypertension services, community screening, mobile or teleophthalmology programs	Non-mydriatic or handheld fundus imaging, automated quality control, and locally validated AI	Prioritize high-risk individuals for eGFR/UACR testing and tele-referral within available capacity	Context-dependent	Transferability across low-cost devices, rates of ungradable images, local calibration, equity, and downstream capacity
**Monitoring in established CKD or kidney replacement therapy**	CKD G3–G5, dialysis, or post-transplant populations in specialist care	OCT/OCTA; selected oximetry, LSFG, or vascular-reactivity measures	Research-oriented assessment of trajectory, residual vascular risk, or treatment response	Exploratory	Test–retest reliability, minimal clinically important change, and evidence that imaging alters management
**General-population screening**	Average-risk asymptomatic adults without an existing retinal imaging pathway	New fundus acquisition and image-only AI screening	Would require confirmatory laboratory testing for a potentially large number of screen-positive individuals	Not ready	Low positive predictive value, risk of over-referral, opportunity cost, fairness, and lack of outcome evidence

**Note:** Translational positions are contextual judgments based on current evidence and workflow readiness, not formal evidence grades. Retinal imaging is intended to enrich risk assessment and prompt confirmatory testing rather than establish CKD independently. **Abbreviations:** AI, artificial intelligence; CKD, chronic kidney disease; CVD, cardiovascular disease; eGFR, estimated glomerular filtration rate; EHR, electronic health record; FP, fundus photography; LSFG, laser speckle flowgraphy; OCT, optical coherence tomography; OCTA, optical coherence tomography angiography; UACR, urinary albumin-to-creatinine ratio; UWF, ultra-widefield imaging.

## Data Availability

No new data were created or analyzed in this study. The complete database search strategies and evidence-selection framework are provided in Supporting Information S1.

## Supplementary Materials

The following supporting information can be downloaded at: https://www.mdpi.com/article/10.3390/jcm15218657/s1, Supporting Information S1. Literature search and evidence-selection framework. Full database search strategies, record counts, evidence-selection framework, evidence prioritization, and author oversight.

## Abbreviations

AI: artificial intelligence; AUC: area under the receiver operating characteristic curve; AVR: arteriolar-to-venular ratio; C-index: concordance index; CKD: chronic kidney disease; DKD: diabetic kidney disease; DN: diabetic nephropathy; DVA: dynamic vessel analysis; eGFR: estimated glomerular filtration rate; EHR: electronic health record; ESRD: end-stage renal disease (retained only when reporting terminology used in the source studies); FAZ: foveal avascular zone; FD: fractal dimension; FFA: fundus fluorescein angiography; FLIO: fluorescence lifetime imaging ophthalmoscopy; FP: fundus photography; GCIPL: ganglion cell–inner plexiform layer; GFR: glomerular filtration rate; HbA1c: glycated hemoglobin; HR: hazard ratio; ICGA: indocyanine green angiography; LSFG: laser speckle flowgraphy; MASLD: metabolic dysfunction-associated steatotic liver disease; NDKD: non-diabetic kidney disease; NDRD: non-diabetic renal disease; NVU: neurovascular unit; OCT: optical coherence tomography; OCTA: optical coherence tomography angiography; pRNFL: peripapillary retinal nerve fiber layer; PROBAST+AI: Prediction Model Risk of Bias Assessment Tool–Artificial Intelligence; RAAS: renin–angiotensin–aldosterone system; RNFL: retinal nerve fiber layer; TRIPOD+AI: Transparent Reporting of a Multivariable Prediction Model for Individual Prognosis or Diagnosis–Artificial Intelligence; UACR: urinary albumin-to-creatinine ratio; UWF: ultra-widefield imaging.
