# Supplementary material for: Multimodal Retinal Imaging for Systemic Microvascular Phenotyping: Chronic Kidney Disease as a Translational Reference Model
Source: J Clin Med. 2026 Nov 6;15(21):8657. doi: 10.3390/jcm15218657 (PMC13763834; doi:10.3390/jcm15218657)
Supplement: Supplementary file 1 [file jcm-15-08657-s001.zip › jcm-4564499-supplementary.pdf]

Supporting Information S1

Literature search and evidence-selection framework

1. Review design and scope

This critical narrative review was designed to synthesize translational evidence linking retinal imaging with chronic kidney disease (CKD), systemic microvascular disease, artificial intelligence (AI), and clinical implementation. It was not prospectively registered and was not designed as a systematic review or meta-analysis. Searches were structured to identify representative and methodologically informative evidence rather than to support a formal pooled effect estimate.

2. Information sources, search coverage, and record counts

PubMed/MEDLINE, Embase (Ovid), and Web of Science Core Collection were searched from database inception. The core database searches were executed on 28 July 2026 and restricted to records published through 27 July 2026. Targeted supplementary searches and reference-list screening were updated through 31 July 2026. No language, human-subject, publication-type, or full-text-availability restrictions were applied at the database-search stage.

Source	Records exported	Internal duplicates removed	Records entering cross-database deduplication
PubMed/MEDLINE	6,290	0	6,290
Embase (Ovid)	5,327	2	5,325
Web of Science Core Collection	1,485	0	1,485
Total	13,102	2	13,100

After cross-database merging and deduplication, 3,379 duplicate records were removed and 9,721 unique records remained. Exact normalized DOI, exact PMID, or exact normalized title (minimum 30 characters) was used for automatic merging; fuzzy-title matches were not merged automatically to reduce false consolidation. Record counts are reported to document database retrieval and deduplication and should not be interpreted as a PRISMA-style study-selection flow. Because this was a critical narrative review, evidence selection was iterative and based on relevance, methodological informativeness, validation quality, and translational importance rather than exhaustive inclusion of every potentially eligible record.

3. Evidence prioritization and author oversight

A Python-based relevance-ranking workflow, developed and checked with AI-assisted coding support (OpenAI Codex), was used solely to prioritize records for author review. It did not independently determine evidence inclusion, conduct detailed appraisal, or generate the evidence synthesis. All decisions regarding evidence selection, detailed appraisal, evidence interpretation, citation, and the use of individual records were made or verified by the authors.

The first author led the detailed reading and selection of literature relevant to the review's central questions. Relevance ranking supported the prioritization of records for reading, while decisions to examine and use individual studies were made iteratively according to their relevance to the manuscript topics and the evidence-selection framework below. Other authors jointly checked the selected literature with the first author. The 9,721 deduplicated records represent the retrieved literature pool, not the number of studies receiving detailed review. Stage-specific screening counts are not reported because the process was an iterative narrative selection rather than a systematic study-selection exercise.

4. Evidence-selection and use framework

Evidence category	Prioritized evidence	Generally excluded from core evidence	Principal use
Human clinical evidence	Peer-reviewed cross-sectional and longitudinal studies; treatment, dialysis, and transplantation studies	Conference abstracts, isolated case reports, or studies without relevant retinal and systemic outcomes	Clinical associations, temporal evidence, treatment sensitivity, and limitations
Guidelines and evidence syntheses	Major clinical guidelines and relevant systematic reviews/meta-analyses	Non-authoritative or non-peer-reviewed summaries	Clinical definitions, standards, and evidence context
AI and prediction studies	Studies with a defined task, cohort composition, quantitative performance, and validation	Models without a relevant retinal input or kidney/systemic outcome; insufficiently	Detection, prediction, validation, and implementation appraisal

	information	described performance	
Mechanistic/preclinical evidence	Selected studies clarifying biological plausibility	Mechanistic material without a clear link to the review framework	Support for shared microvascular and neurovascular mechanisms
Methodological evidence	Reporting, risk-of-bias, calibration, fairness, decision-curve, workflow, and implementation frameworks	Methodological material unrelated to the intended clinical pathway	Assessment of translational readiness

Mechanistic and preclinical evidence was included selectively when it clarified biological plausibility. Cardiovascular and cardiometabolic evidence was prioritized as the principal comparative context, whereas liver-, gut-, neurodegenerative-, and immune-inflammatory evidence was used primarily to inform contextual interpretation, phenotypic heterogeneity, and potential effect modification. Main-text Tables 2 and 3 present illustrative selections from the reviewed literature rather than exhaustive inventories or formally ranked sets of studies. Table 2 brings together clinical evidence across imaging modalities, kidney phenotypes, and study designs, illustrating both reported associations and the extent of longitudinal or incremental predictive evidence. Table 3 presents retinal AI models across clinical tasks and validation settings, highlighting their comparators, performance, and translational limitations. These organizing considerations describe the purpose and composition of the tables; they were not applied as a prespecified scoring system for study inclusion.

5. Database search strategies

PubMed/MEDLINE

```
(
  "Retina"[Mesh] OR "Retinal Vessels"[Mesh] OR "Choroid"[Mesh] OR "Fundus Oculi"[Mesh]
  OR "Tomography, Optical Coherence"[Mesh] OR "Fluorescein Angiography"[Mesh]
  OR retina[tiab] OR retinal[tiab] OR retinovascular[tiab] OR "retinal vascular"[tiab]
  OR "retinal microvascular"[tiab] OR "retinal microcirculation"[tiab] OR choroid*[tiab]
  OR fundus[tiab] OR ophthalmoscop*[tiab] OR "optical coherence tomograph*"[tiab]
  OR OCTA[tiab] OR "angio-OCT"[tiab] OR "retinal photograph*"[tiab]
  OR "retinal imag*"[tiab] OR "retinal oximetr*"[tiab]
  OR "laser speckle flowgraph*"[tiab] OR "dynamic vessel analys*"[tiab]
)
AND
(
  "Renal Insufficiency, Chronic"[Mesh] OR "Kidney Diseases"[Mesh]
  OR "Kidney Function Tests"[Mesh] OR "Glomerular Filtration Rate"[Mesh]
  OR "Albuminuria"[Mesh] OR "Proteinuria"[Mesh] OR "Renal Dialysis"[Mesh]
  OR "Kidney Transplantation"[Mesh] OR "Diabetic Nephropathies"[Mesh] OR "Cystatin C"[Mesh]
  OR "chronic kidney disease*"[tiab] OR "chronic renal disease*"[tiab]
  OR "chronic renal insufficien*"[tiab] OR "kidney dysfunction"[tiab]
  OR "renal dysfunction"[tiab] OR "kidney function"[tiab] OR "renal function"[tiab]
  OR eGFR[tiab] OR "estimated glomerular filtration rate"[tiab]
  OR albuminuria[tiab] OR proteinuria[tiab] OR "urinary albumin"[tiab]
  OR "end-stage kidney disease"[tiab] OR "end stage renal disease"[tiab]
  OR dialysis[tiab] OR hemodialysis[tiab] OR haemodialysis[tiab]
  OR "kidney transplant*"[tiab] OR "renal transplant*"[tiab]
  OR "diabetic kidney disease"[tiab] OR "diabetic nephropath*"[tiab]
)
AND ("1900/01/01"[Date - Publication] : "2026/07/27"[Date - Publication])
```

Archived high-sensitivity query; 6,290 records were exported.

Embase (Ovid)

```
1. (retinovascular or (retina$ adj3 (vascul$ or microvascul$ or microcirculat$ or vessel$ or photograph$ or imag$ or thickness or perfusion or oximetr$)) or fundus or OCTA or angio-OCT or (optical coherence tomograph$ adj3 (retina$ or choroid$ or fundus)) or laser speckle flowgraph$ or dynamic vessel analys$ or (choroid$ adj3 (imag$ or thickness or vascular$ or perfusion))).ti,ab,kw.
2. exp retinal blood vessel/ or exp choroid/ or exp optical coherence tomography/ or exp fluorescein angiography/
3. 1 or 2
4. (chronic kidney disease$ or chronic renal disease$ or chronic renal insufficien$ or kidney dysfunction or renal dysfunction or kidney function or renal function or eGFR or estimated glomerular filtration rate or albuminuria or proteinuria or urinary albumin or (end adj2 stage adj2 (kidney or renal) adj2 disease$) or ESKD or ESRD or dialysis or hemodialysis or haemodialysis or kidney transplant$ or renal transplant$ or diabetic kidney disease or diabetic nephropath$).ti,ab,kw.
```

5. exp chronic kidney disease/ or exp glomerular filtration rate/ or exp albuminuria/ or exp proteinuria/ or exp hemodialysis/ or exp kidney transplantation/ or exp diabetic nephropathy/
6. 4 or 5
7. 3 and 6

The final set retrieved 5,327 records; two internal duplicates were removed before database merging.

Web of Science Core Collection

#1 TS=(retinovascular OR (retina* NEAR/3 (vascul* OR microvascul* OR microcirculat* OR vessel* OR photograph* OR imag* OR thickness OR perfusion OR oximetr*)) OR fundus OR OCTA OR "angio-OCT" OR ("optical coherence tomograph*" NEAR/3 (retina* OR choroid* OR fundus)) OR "laser speckle flowgraph*" OR "dynamic vessel analys*" OR (choroid* NEAR/3 (imag* OR thickness OR vascular* OR perfusion))) 110,863 records
#2 TS=("chronic kidney disease*" OR "chronic renal disease*" OR "chronic renal insufficien*" OR "kidney dysfunction" OR "renal dysfunction" OR "kidney function" OR "renal function" OR eGFR OR "estimated glomerular filtration rate" OR albuminuria OR proteinuria OR "urinary albumin" OR ((end-stage OR "end stage") NEAR/2 (kidney OR renal) NEAR/2 disease*) OR ESKD OR ESRD OR dialysis OR hemodialysis OR haemodialysis OR "kidney transplant*" OR "renal transplant*" OR "diabetic kidney disease" OR "diabetic nephropath*") 763,819 records
#3 #1 AND #2 1,485 records exported

The archived Web of Science session recorded 110,863 records for the retinal/choroidal imaging concept, 763,819 records for the CKD/renal-function concept, and 1,485 records in their intersection. In the original platform session, these appeared as sets #3, #5, and #6 (#3 AND #5), respectively; they are renumbered below as #1-#3 for reporting clarity.

The 1,485 records in the final Web of Science set were exported for cross-database deduplication. No language, human, document-type, or full-text filter was applied.

6. Abbreviations

AI, artificial intelligence; CKD, chronic kidney disease; DOI, digital object identifier; eGFR, estimated glomerular filtration rate; ESKD, end-stage kidney disease; ESRD, end-stage renal disease; OCTA, optical coherence tomography angiography; PMID, PubMed identifier; SANRA, Scale for the Assessment of Narrative Review Articles.
